# Highly efficient light-gated COF membrane for precise multistage molecular separation

**DOI:** 10.1126/sciadv.adz1929

**Published:** 2026-03-13

**Authors:** Liyong Zhai, Shuaiqi Gao, Linlin Guo, Shuangjiang Luo, Zhiyong Li, Huiyong Wang, Suojiang Zhang, Jianji Wang

**Affiliations:** ^1^ Key Laboratory of Green Chemical Media and Reactions (Ministry of Education), Collaborative Innovation Center of Henan Province for Green Manufacturing of Fine Chemicals, School of Chemistry and Chemical Engineering, Henan Normal University, Xinxiang 453007, Henan, P. R. China.; ^2^ CAS Key Laboratory of Green Process and Engineering, Beijing Key Laboratory of Ionic Liquids Clean Process, State Key Laboratory of Multiphase Complex Systems, Institute of Process Engineering, Chinese Academy of Sciences, Beijing 100190, P. R. China.; ^3^ College of Chemistry and Molecular Sciences, Longzihu New Energy Laboratory, Henan University, Zhengzhou 450000, P. R. China.

## Abstract

Membrane technology is crucial for the separations in chemical and biochemical industries because of its flexibility and reliability. However, conventional multistage separation requires multiple membranes with different pore sizes, leading to low efficiency and high energy consumption. Inspired by the light-responsive behavior of stomata in natural leaves, we successfully fabricated a highly efficient light-gated covalent organic framework (COF) membrane by using click reaction to incorporate 53.5 weight % azobenzene into the nanochannels, where the pore size of this membrane could be dynamically switched between 0.73 and 0.93 nanometers because of the reversible trans-cis isomerization of azobenzene. Notably, the light-gated COF membrane enabled efficient multistage separations of cannabidiol oil (a high-value pharmaceutical), limonene, and chlorophyll in hemp oil as well as gold, silver, and iron ions in simulated natural ores. The separation process remained stable over 100 trans-cis-trans cycles as a result of the formation of stable triazole bonds. Thus, this work provides an instructive strategy to develop light-gated COF membranes for precise molecular separations.

## Teaser

Light-gated COF membrane enables effective and continuous sieving in precise multistage molecular separation for at least 12 days.

## INTRODUCTION

With gradual deterioration of the global climate and depletion of the fossil resources, the concept of sustainable development is becoming increasingly important and has been included in various regulations by a number of countries and organizations (*1*). The chemical industry plays an indispensable role in modern civilization of our society from daily life to planetary exploration (*2*). However, it is still the biggest energy consumption and carbon emission sector in the world until now. The main reason is that traditional separation processes, such as distillation, adsorption, extraction, and crystallization, consume about 60% of the world’s total industrial energy consumption (*3*) and lead to high carbon emission and secondary pollution, which is a great obstacle to sustainable development. Membrane separation is an effective technology for separation and recycling of multicomponent molecules, affording advantages such as low energy consumption, low carbon emission, high environmental safety, and economic feasibility (*4*, *5*). Nevertheless, the separation efficiency of traditional membranes is usually low because of the lack of well-ordered and adjustable pores, and two or more membranes have to be used for the separation of multiple components with different molecular sizes, which causes high energy and capital consumption. Therefore, it is of great importance to create smart membranes whose pore size can be tuned in real time for the effective separation of different sizes of molecules.

**Fig. 1.**
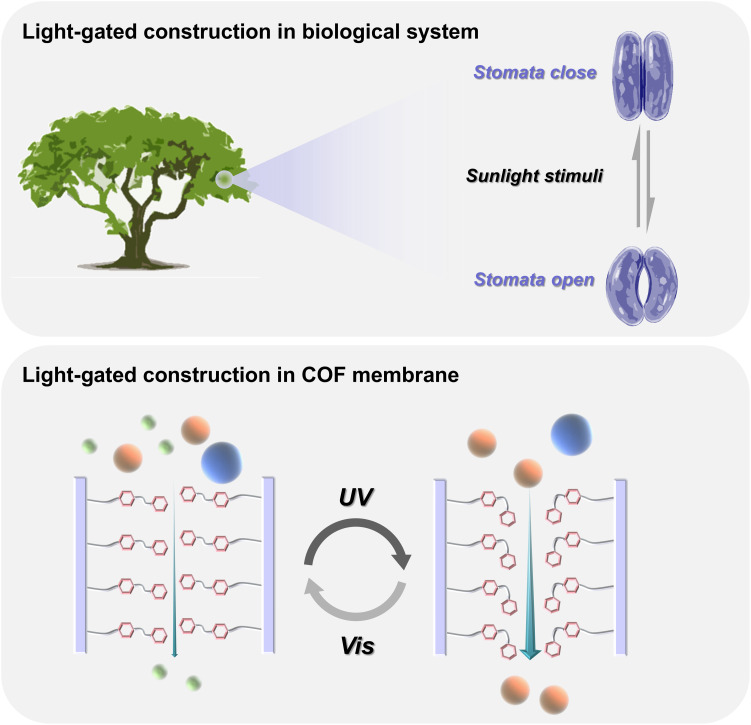
Illustration of bioinspired light-gated pore channels. Schematic of the stomata with open and closed pores under external stimuli (**top**); schematic of artificial light-gated channels for molecular separations (**bottom**).

In nature, many biological channels can be modulated to respond for an external stimulus. Stomata are a typical example (*6*), which can be used to adjust pore size in leaves and stalks under light irradiation (Fig. 1). This gives us an excellent inspiration for the use of light to regulate the pore size of membranes. Specifically, the stomata are formed by two guard cells, which are usually distributed evenly on the leaf epidermis and run vertically across the leaf surface (*7*). In the absence of light, the guard cells swell with water, keeping the stomata closed, while in the presence of light, the guard cells shrink by losing water, leading to the stomata opening.

Inspired by this interesting biological process, two key points must be considered for the construction of efficient multipurpose photoresponsive membranes: One is the fast and stable pore size response to light, and the other is the highly ordered pores perpendicular to the membrane. It is known that azobenzene groups, with two phenyl rings separated by an azo bond (─N═N─) (*8*), can enable trans-to-cis isomerization under ultraviolet (UV) light and get back to cis-to-trans isomerization under visible (vis) light irradiation (*9*). Comparing the configuration of cis and trans isomers of azobenzene, the molecular size can be changed from planar *trans*-azobenzene (~9 Å) to a nonplanar cis isomer (~6 Å) with a dipole moment change from 3.1 D to 0 (*10*). This provides a promising pore wall material for the synthesis of smart membranes with photoswitching pores. However, it is not possible for traditional membranes to meet the requirement of these key points because of the lack of light responsiveness and uniform nanoscale channels. Thus, the exploration of light-responsive membrane materials featuring highly ordered nanochannels is of great significance.

Covalent organic frameworks (COFs) are a novel class of highly crystalline porous materials and show the advantages of tunable pore size, large surface area, ease of modification, and superior physical and chemical stability (*11*, *12*). Combined with azobenzene modification and isomerization, these distinctive superiorities may offer promising applications in light-responsive smart membranes. Recently, several efforts have been made to fabricate continuous photoresponsive COF membranes. In this context, Yin and co-workers (*13*) engineered a light-responsive COF membrane through amidation reaction for the discrimination of K^+^ and Al^3+^, which exhibited a grafting efficiency of ~80%, a selectivity of 6000, and at least five light switching cycles. Ren and co-workers (*14*) fabricated smart COF membranes decorated with photoresponsive azobenzene derivatives through sulfur-fluoride exchange reaction to sieve different metal ions, and the selectivity values were 17.9 for K^+^/Li^+^ and 24.9 for Li^+^/Mg^2+^ within 10 cycles of reversible closing and opening of nanochannels. After careful analysis of the above results, it is found that the high content and stability of the light-responsive units grafted on the COFs are both crucial for practical applications of smart membranes but still a great challenge because of the lower efficient grafting reaction and the weaker bonding of azobenzene with the anchoring group in the COF, which may limit its application scope and scenarios. This is the main reason why the separation of multiple components by the light-responsive COF membrane is rarely reported.

In this work, inspired by the light-responsive behavior of stomata in natural leaves, we focused on the design and synthesis of biomimetic light-gated COF membranes with high stability and high azobenzene content. Considering the fact that click reaction is a useful approach for postmodification because of its high efficiency and easy operation procedures (*15*), it was used to graft azide-azobenzene onto alkyne sites on the wall of nanochannels as dangling groups through the formation of stable triazole bonds in the imine-linked COF membranes. The successful grafting of azobenzene was confirmed by various characterizations, and the grafting efficiency of azobenzene was 98.5%. Upon alternating UV and vis light irradiation, highly efficient and reversible trans-cis isomerization of azobenzene in the COF membranes was achieved with a conversion efficiency of 47.1%. Because of the different configurations of trans and cis isomers, the pore size of the membranes was precisely tuned within the range between 0.73 and 0.93 nm, thereby providing the opportunity for the sieving of different sizes of molecules. As a result, the optimal COF membrane exhibited high potential for sieving cannabidiol (CBD) oil, limonene, and chlorophyll in the hemp oil as well as gold, silver, and iron ions in natural ores with excellent selectivity and durability.

## RESULTS

### Synthesis and characterizations of the COF membranes

**Fig. 2.**
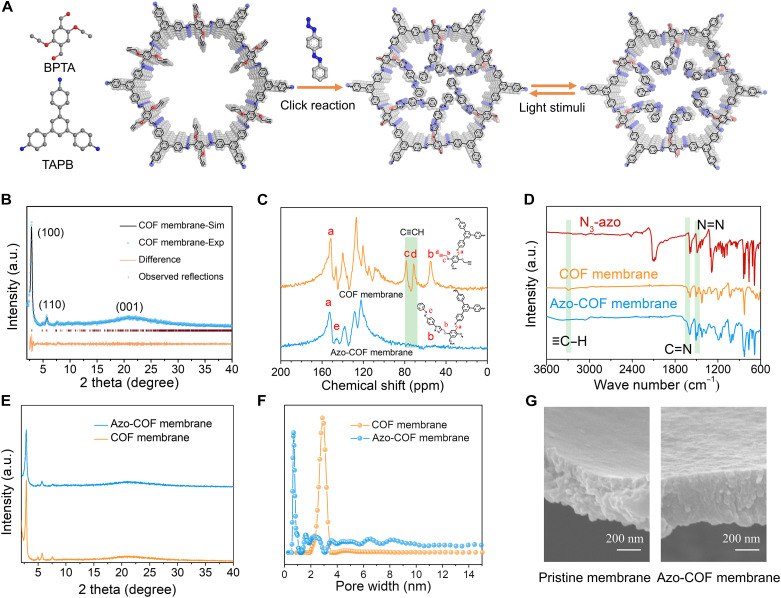
Synthesis and characterization of the COF membrane. (**A**) Synthesis of the COF and Azo-COF membranes. (**B**) Experimental and simulated PXRD patterns of the COF membrane. a.u., arbitrary units. (**C**) Solid-state ^13^C cross-polarization magic-angle-spinning NMR spectra of the COF and Azo-COF membranes. (**D**) FTIR spectra of the pristine and azobenzene-grafted COF membranes. (**E**) PXRD patterns of the azobenzene-grafted COF membrane compared to the pristine membrane. (**F**) Pore size distribution of the pristine and azobenzene-grafted COF membranes based on the N_2_ adsorption/desorption isotherm. (**G**) Cross section of the SEM image for the pristine (left) and azobenzene-grafted (right) COF membranes.

Here, 2,5-bis(2-propynyloxy)terephthalaldehyde (BPTA) and 1,3,5-tris(4-aminophenyl)benzene (TAPB) were used to synthesize COF membranes with free acetenyl groups (click reaction sites) on the wall of their nanochannels (Fig. 2A) at the ionic liquid (IL)-H_2_O interface. In doing so, we first prepared the 1-decyl-3-methylimidazolium bis[(trifluoromethane)sulfonyl]imide ([C_10_Mim][Tf_2_N]) IL (see details in the Supplementary Materials and fig. S1), which was then combined with water to construct the IL-H_2_O interface for the synthesis of the BPTA-TAPB COF membrane with *p*-toluenesulfonic acid as a catalyst (fig. S2). As reported previously (*16*), the diffusion of BPTA monomer in the IL was effectively slowed down by the high viscosity of the IL, and the transport of TAPB in water was significantly controlled by hydrogen bonding between TAPB and the catalyst. Thus, highly controlled polymerization, growth, and assembly of the crystal COF were achieved at the IL-H_2_O interface to fabricate a smooth, continuous, and freestanding membrane (named the BPTA-TAPB COF membrane) at room temperature after 72 hours (fig. S3A). Furthermore, defect-free and continuous COF membranes with the diameters of 25, 30, and 50 mm were also fabricated by adjusting the sectional area of vessels (fig. S3B).

The structure and crystalline characteristics of the COF membrane were first characterized by powder x-ray diffraction (PXRD) (Fig. 2B), where the peak at 2.87° was assigned to the (100) facet (*17*), indicating the regularly ordered hexagonal lattice and highly crystalline nature. The broad peak at 21.2° was attributed to the (001) facet (*18*), while the peak at 5.72° was ascribed to the (110) facet (*19*), which matches well with the simulated pattern of an AA stacking mode. The peak at 2.87° showed a shift compared with the previously reported position (*15*), which might be attributed to the quite different synthesis conditions of the COFs (*20*). Moreover, the unit cell parameters were calculated to confirm the structure of the COF membrane (fig. S4), and the results were given as *a* = 37.96 Å, *b* = 37.41 Å, *c* = 3.56 Å, α = 90°, β = 90°, and γ = 120°, with low factors of *R*_wp_ = 3.04% and *R*_p_ = 2.27%. Compared with TAPB and BPTA monomers (fig. S5), the Fourier transform infrared (FTIR) spectrum of the COF membrane exhibited several features: The peak at 1613 cm^−1^ was ascribed to the stretching vibration of ─C═N in the skeleton of imine linked COF, the peaks at 3286 and 2117 cm^−1^ were contributed from the vibration of C≡C and CC-H in alkynes (*21*) on the porous wall of the membrane, while the disappeared peaks at 1675 cm^−1^ and 3400 to 3200 cm^−1^ were attributed to the stretching vibration of C═O in the BPTA monomer and N─H in the TAPB monomer (*22*). These results indicate that we had successfully synthesized the aimed COF membrane. Furthermore, the characteristic signal at 152 parts per million (ppm) in solid-state ^13^C cross-polarization magic-angle-spinning nuclear magnetic resonance (NMR) was assigned to the ─C═N bond in the skeleton of imine-linked COF (*23*), confirming the formation of imine-based COF (Fig. 2C). In the high-resolution N 1s spectrum, the peaks at 400.3 and 399.8 eV were ascribed to ─C─N and ─C═N in the skeleton of the membrane (fig. S6) (*24*). For the studies on the porosity of the COF membrane, the N_2_ adsorption-desorption isotherm was measured, from which the Brunauer-Emmett-Teller (BET) surface area was determined to be 1649 m^2^ g^−1^ (fig. S7), and the pore size was calculated to be mainly at 2.81 nm (Fig. 2F) using nonlocal density functional theory. The morphology and thickness of the COF membrane were analyzed by scanning electron microscopy (SEM), which suggest that the membrane surface was smooth (fig. S8), and the thickness was about 400 nm for the BPTA-TAPB COF membrane (Fig. 2G). Moreover, transmission electron microscopy (TEM) images and selected area electron diffraction patterns (fig. S9) also revealed the high crystallinity of the as-synthesized COF membrane.

Next, *p*-azido-azobenzene (Azo) with azido groups was synthesized and characterized (figs. S10 and S11) and then grafted on the pore wall of the COF membrane by click reaction through the formation of triazole bonds between alkyne and azido to obtain a light-gated COF membrane, which was named the Azo-COF membrane. Then, the chemical structure of the Azo-COF membrane was first characterized by FTIR spectroscopy. It was found that the vibration peaks of C≡C and CC-H at 3286 and 2117 cm^−1^ disappeared in the COF membrane (*25*), but a new characteristic peak assigned to the vibration of N═N was observed at about 1489 cm^−1^, indicating the complete reaction between free alkyne and azido. Meanwhile, the stretching vibration of C═N at 1613 cm^−1^ was well maintained in the COF membrane (Fig. 2D) (*14*). The solid-state ^13^C NMR spectrum indicates that the characteristic peaks of the alkynyl fragment at 80 and 73 ppm also disappeared after grafting Azo groups, which means that the reaction between the alkyne and azido was completed (Fig. 2C). The new peak at 144 ppm was ascribed to aromatic carbons in the C─N═N─C linkage, and the peak at 152 ppm represented ─C═N in the skeleton of the imine-based membrane, which indicate that the COF membrane skeleton completely remained (*13*). The high-resolution N 1s spectrum showed a new peak at 401.8 eV, corresponding to the N═N bond in the grafted azobenzene groups (fig. S12) (*24*). Moreover, the UV-vis diffuse reflectance spectrum revealed that the absorption wavelength exhibited a shift to the vis light region, demonstrating the successful introduction of azobenzene groups (fig. S13). The above characterizations verified the precise grafting of the azobenzene units onto pore nanochannels of the COF membrane by click reaction. The grafting rate of azobenzene was determined by using elemental analysis, and the maximum grafting rate of azobenzene was calculated to be 53.5 wt % in the Azo-COF membrane (table S1), which is very close to the theoretical maximum content of 53.7 wt %. The atomic percentage of C, N, and O determined from x-ray photoelectron spectroscopy and energy-dispersive x-ray spectroscopy mapping was in good agreement with the 53.5% grafting rate of azobenzene in the Azo-COF membrane determined from elemental analysis (table S2), confirming the reliability of the elemental analysis approach. Furthermore, the ^1^H NMR spectrum of the concentrated acid–digested Azo-COF membranes also showed a 98.5% grafting efficiency, corresponding to the 53.0 wt % grafting rate, which is very close to the value (53.5 wt %) obtained from element analysis (fig. S14). X-ray photoelectron spectroscopy depth profile analysis was also performed, which indicated a homogeneous chemical environment from the surface to the interior of the membrane, demonstrating an even distribution of the grafted azobenzene groups without significant depth-dependent gradients (fig. S15).

It is worth noting that the Azo-COF membrane showed the same PXRD diffraction pattern as the BPTA-TAPB COF membrane at 2.87° (Fig. 2E), evidencing that the crystalline lattice structure of the pristine COF membrane was still maintained, although the diffraction intensity was reduced because of the possible slight disorder of the flexible Azo units on the wall of the nanochannels (*15*, *25*). However, this did not mean a decreased ordering of the nanochannels in the membrane (*26*). According to the N_2_ adsorption and desorption isotherms (fig. S16), the BET surface area and pore size were determined to be 78 m^2^ g^−1^ and 0.73 nm, respectively, for the Azo-COF membrane (Fig. 2F). Obviously, the BET surface area and pore size of the membrane were substantially decreased after grafting reaction, which might be ascribed to the introduction of azobenzene units on the wall of nanopores. Such a dramatic decrease indicates that azobenzene was distributed within the pores of the COF membrane and occupied a certain volume of the pores. Compared with the N_2_ molecule, CO_2_ has a smaller kinetic diameter (0.33 nm versus 0.36 nm), higher kinetic energy, and higher diffusion capacity. Thus, the CO_2_ adsorption isotherm of the Azo-COF membrane was also determined at 195 K to examine the porous characteristics of the Azo-COF membrane (fig. S17). The BET surface area and pore size were found to be 254 m^2^ g^−1^ and 0.74 nm, respectively, indicating that the porous characteristics remained to a certain extent after grafting of azobenzene units. It was also found from SEM images that there was no obvious change in the surface topography of the membrane (fig. S18), and the membrane thickness was almost unchanged (Fig. 2G). In addition, the structure of the membrane was also investigated by the TEM image and selected area electron diffraction pattern (fig. S19), revealing numerous micropores and the high crystallinity of the Azo-COF membrane.

### Photoisomerization of azobenzene in the Azo-COF membrane

**Fig. 3.**
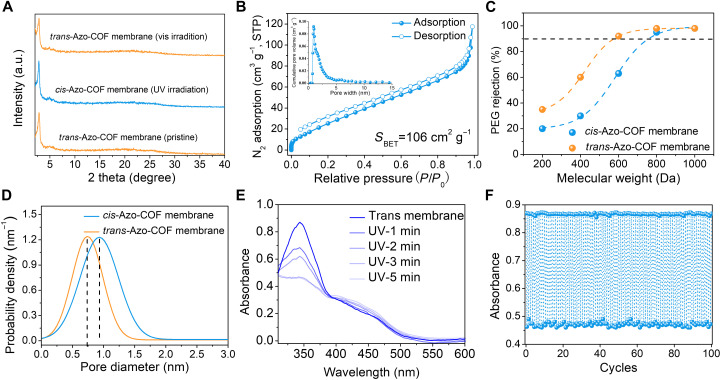
Photoisomerization of azobenzene in the Azo-COF membrane. (**A**) PXRD patterns of the Azo-COF membrane before and after UV light irradiation. (**B**) N_2_ adsorption/desorption isotherm and corresponding pore size distribution after UV light irradiation. STP, standard temperature and pressure. (**C**) PEG rejection curves of the *trans*/*cis*-Azo COF membranes. (**D**) Pore size distribution of the *trans*/*cis*-Azo COF membranes calculated from MWCO. (**E**) UV-vis spectra of the Azo-COF membrane in DMF with UV light irradiation. (**F**) Photoisomerization cycles of the Azo-COF membrane dispersed in DMF with alternative UV and vis light irradiation.

The photoisomerization of azobenzene in the nanochannels of the Azo-COF membrane was studied under UV and vis light irradiation. In the fresh Azo-COF membrane, azobenzene was at the trans status, and the Azo-COF membrane was denoted as the *trans*-Azo-COF membrane. After irradiation by UV light, trans-to-cis transformation of azobenzene was achieved to acquire the *cis*-Azo-COF membrane. It can be seen from Fig. 3A that the crystallinity of *trans*-Azo-COF and *cis*-Azo-COF membranes was almost the same and not affected by the trans/cis photoisomerization of azobenzene in the membranes, which ensures the framework stability during isomerization. Then, N_2_ adsorption and desorption isotherms were determined to verify the variation of BET surface area and pore size in the photoisomerization of the *trans*/*cis*-Azo-COF membranes. It was found that compared to the *trans*-Azo-COF membrane, the BET surface area and pore size of the *cis*-Azo-COF membrane increased (78 m^2^ g^−1^ versus 106 m^2^ g^−1^ and 0.73 nm versus 0.91 nm, respectively) (Fig. 3B), which could be explained by the more accessible pores caused by the cis isomer of azobenzene groups in the Azo-COF membrane (*27*). Furthermore, molecular weight cutoff (MWCO) measurement was conducted by performing the rejection experiments of neutral polyethylene glycol (PEG) molecules in water (*28*) and a series of standard dyes with known molecular weight in ethanol (*29*) to account for the pore size distribution of the light-responsive *trans*/*cis*-Azo-COF membranes. As shown in the PEG rejection curves (Fig. 3C), the *trans*/*cis*-Azo-COF membranes displayed MWCO values of around 572 and 755 Da, respectively, and the aperture pore size was calculated to be 0.73 and 0.93 nm on the basis of the solute Stokes diameter (Fig. 3D) (*30*). These values are very close to the pore size distribution obtained from the analysis of the N_2_ adsorption-desorption isotherms. Similarly, the MWCO values of the *trans*/*cis*-Azo-COF membranes were determined to be around 503 and 648 Da from the standard dyes in ethanol, and the aperture pore size was 0.74 and 0.91 nm, respectively (fig. S20). Thus, the pore size could be precisely switched at an angstrom level by UV-vis light irradiation, providing the possibility for the separation of different sizes of molecules by using only one light-gated membrane.

The trans/cis photoisomerization of azobenzene groups was also investigated by dispersing the N_3_-azo monomer and Azo-COF membrane in *N*,*N*′-dimethylformamide (DMF) (*14*, *31*). The UV-vis spectrum shows two characteristic absorption peaks at 332 and 440 nm (Fig. 3E and fig. S21), indicating π-π* and n-π* transitions of the azobenzene groups, respectively. After exposure to UV light, the intensity of the π-π* absorption peak at 332 nm reduced significantly, while that of the n-π* absorption peak at 440 nm enhanced slightly. However, upon irradiation with vis light, the peaks corresponding to the π-π* and n-π* transitions could be recovered to the initial intensity, indicating a good reversibility of trans/cis photoisomerization. The trans-to-cis photoisomerization efficiency of the Azo-COF membrane could reach 47.1% (Fig. 3E). It was noted that the dangling azobenzene groups twist away from the COF plane to form a propeller arrangement, leading to nonplanar *cis*-azobenzene with a C─N_azo_═N_azo_─C angle of 37° (*32*). Notably, the reversible trans-cis-trans photoisomerization of azobenzene exhibited a fast response (~3 min), a high trans-cis isomerization rate (47.1%), and highly efficient photoisomerization reversibility, which could be continuously operated for at least 100 cycles without obvious degradation and changes in the membrane structure (Fig. 3F and fig. S22). Photoisomerization switching also led to the change of surface wettability (fig. S23), and the contact angle decreased from 108° to 98° when *trans*-azobenzene was transferred to the cis isomer because the dipole of the cis isomer was higher than that of the trans isomer (*31*), resulting in a more hydrophilic surface.

### Light-gated COF membrane for graded molecular sieving

**Fig. 4.**
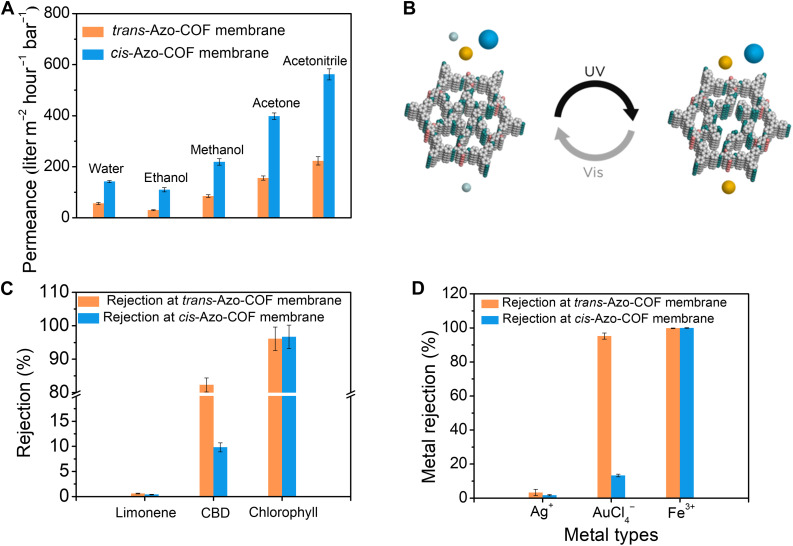
Light-gated separation performance of the Azo-COF membranes. (**A**) Permeance of protic and aprotic solvents through the *trans*/*cis*-Azo-COF membranes. (**B**) Schematic of light-gated molecular gradient separation through *trans*/*cis*-Azo COF membranes. (**C**) Comparison of the rejection rate of CBD and its competition molecules through the *trans*/*cis*-Azo-COF membranes. (**D**) Comparison of the rejection rate of different ions through the *trans*/*cis*-Azo-COF membranes.

Before the separation of multicomponents, a series of aprotic solvents (e.g., acetonitrile and acetone) and protic solvents (e.g., water, methanol, and ethanol) were used in a homemade dead-end mode stirred cell (figs. S24 and S25) to estimate the solvent permeance of the Azo-COF membrane. As depicted in Fig. 4A, the *trans*-Azo-COF membrane demonstrated permeance values of 56, 85, 30, 156, and 223 liters m^−2^ hour^−1^ bar^−1^ for water, methanol, ethanol, acetone, and acetonitrile, respectively. Compared with protic solvents, aprotic solvents exhibited higher permeance performance because of their weak hydrogen bonding within the membrane (*19*). After irradiation by UV light, the as-obtained *cis*-Azo-COF membrane showcased the permeance values of 142, 219, 110, 398, and 562 liters m^−2^ hour^−1^ bar^−1^ for water, methanol, ethanol, acetone, and acetonitrile, respectively, which were 2.5 to 3.7 times that of the *trans*-Azo-COF membrane, owing to the opening pores of the *cis*-Azo-COF membrane.

On account of the porosity switching behavior of the Azo-COF membrane, the membrane was used to separate multiple component mixtures under photostimuli at room temperature. In principle, many different molecules can be separated with the light-gated membrane reported here by using the difference in molecular size to achieve a typical separation and purification purpose. As shown in fig. S26, reversible pore size modulation (0.73 to 0.93 nm) was achieved for the Azo-COF membrane under light switching, which enables precision separation of molecules/ions with a subangstrom resolution by a single-membrane multistage separation strategy. As a proof of the concept, two typical application scenarios were explored below.

The first application scenario is the multistage sieving of CBD, limonene, and chlorophyll in hemp oil. CBD is a high-value pharmaceutical and has vital significance in the treatment of anxiety, depression, and cancer diseases (*33*). It is greatly demanded in recent years with a considerable global market of 2 billion US dollars by 2022 (*34*). Typically, CBD can be extracted from the natural hemp leaves (*35*). For this purpose, the hemp leaves must be crushed to increase the contact area with the extracting solvents such as ethanol and then separated and purified to obtain the final target product. However, the current state-of-the-art processes for the purification of CBD mainly depend on the complicated and energy-intensive methods such as chromatography and recrystallization (*36*). Membranes may be an alternative for the purification of CBD, and critical to this opportunity is the accurate differentiation of CBD from other solutes of different dimensions in the extracts. Thus, on the basis of the practical separation scenario of CBD, we simulated the extract to include three classes of molecules: larger-sized molecule of chlorophyll (>400 g mol^−1^, 1.56 nm by 1.17 nm), middle-sized molecules of CBD and its derivatives (300 to 400 g mol^−1^, 1.31 nm by 0.65 nm), and smaller molecule of limonene (<300 g mol^−1^, 0.25 nm by 0.62 nm) (fig. S27). As mentioned above, the pore size of our light-gated Azo-COF membrane could be switched between 0.93 nm (open state) and 0.73 nm (close state), which is very beneficial for the direct sieving of CBD from the extracts.

First, CBD was synthesized according to the procedures reported previously (*37*) and proven by ^1^H NMR spectroscopy (fig. S28). Before the separation process, we immersed the *trans*-Azo-COF membrane in the feed solution for 24 hours to examine whether the membrane could absorb feed solutes. As shown in fig. S29, the adsorption of limonene, CBD, and chlorophyll was negligible with the concentration changes of only 0.4, 0.3, and 0.3%, respectively, in the feed solution; thus, the adsorption effect was excluded. Then, the rejection and permeation performance of each kind of molecules in ethanol was investigated at the concentration of 10 mg/liter for chlorophyll, CBD, and limonene. As can be seen from the UV-vis spectra (Fig. 4C and figs. S30 to S32), limonene with a smaller molecular size was allowed to freely permeate through the *trans*-Azo-COF membrane (with a close-state pore of 0.73 nm), and ~17.7% CBD could also permeate the membrane, but the larger-sized chlorophyll would be completely rejected into the feed side with a rejection rate of 98.7%. Notably, after irradiation with UV light, the pore of the membrane was at the open state so that CBD could permeate the *cis*-COF membrane (with an open-state pore of 0.93 nm) easily with the permeation of 90.2% because of its smaller molecular size compared with the *cis*-state pore width. However, the chlorophyll with a larger size was almost fully excluded with the rejection rate of 98.8%. Consequently, it is expected that the membrane could sieve limonene, CBD, and chlorophyll with a few CBD loss.

Encouraged by this result, we conducted size-selective separation using mixed chlorophyll, CBD, and limonene as a feed solution with the concentration of 10 mg/liter for each component. As shown in fig. S33, the rejection rates of limonene, CBD, and chlorophyll were 0.9, 85.3, and 98.1% by the *trans*-Azo-COF membrane with a closing pore, respectively, that is, almost all the limonene passed through the membrane and was first collected, while CBD and chlorophyll remained in the feed solution. By contrast, the rejection rates of CBD and chlorophyll were 8.9 and 98.5% by the *cis*-Azo-COF membrane with an opening pore, respectively, which indicate that CBD and chlorophyll were collected in the permeate solution and feed solution, respectively. Furthermore, the high-performance liquid chromatography method was used to confirm the quantification of mixed solution separation, and good agreement was observed between high-performance liquid chromatography and UV-vis measurements (table S3), demonstrating the feasibility of quantification by UV-vis spectroscopy. Therefore, the enrichment of three components in the mixed solution could be achieved by effective sieving using only one light-gated COF membrane with high enrichment factors (fig. S34) of limonene (85.7%), CBD (96.9%), and chlorophyll (91.5%). Combined with the pore size distribution of the *trans*/*cis*-Azo-COF membranes and the molecular size of the extracts, the outstanding separation performance is dominantly attributed from the size exclusion effect. The molecule with an effective size smaller than the pore width of the membrane could freely permeate the membrane, while that with a larger size was rejected into the feed side (Fig. 4B).

The second application scenario is the multistage sieving of gold, silver, and iron ions from a simulated natural ore. Gold, one of the nonrenewable precious metals, has excellent advantages of high conductivity, high ductility, and strong corrosion resistance, which has been extensively used in jewelry, semiconductors, catalysis, medicine, and other industries (*38*, *39*). Natural ore is the most important source of gold for meeting the demands of rapid development of our civilized society. However, the conventional techniques for the separation of gold from its associated components, such as cyanide leaching (*40*), thiourea leaching (*41*), and thiosulfate leaching (*42*), not only require high energy consumption but also generate hazardous wastes (*43*). To address this problem, it is essential to seek a green and low-energy strategy to separate gold from natural ores. The Azo-COF membrane reported in this work could also achieve the goal for the separation of gold from natural ores by light-gating pore sieving.

It is well known that gold always coexists with other metals such as silver and iron in natural ores, resulting in low selectivity in separation (*44*) because of the interference of other metals. In the typical Au-Ag-Fe ores, the Au content is usually from 5 to 50 ppm, and the Ag content is in the range of 2 of 40 ppm, whereas Fe is a major metal in the associated ores with the content range of 0.1 to 20 wt % (*45*, *46*). Therefore, 30 ppm of AgNO_3_, 30 ppm of HAuCl_4_, and 1000 ppm of Fe(NO_3_)_3_ in water were simulated to investigate the multistage separation performance of the Azo-COF membrane by alternating UV and vis light irradiation. As shown in fig. S35, the ion adsorption effect was also negligible with concentration changes of only 0.5, 0.6, and 0.3% for Ag^+^, AuCl_4_^−^, and Fe^3+^, respectively, in the feed solution. In the test of a single type of metal ion, the *trans*-Azo-COF membrane with pores at the closing state demonstrated rejection rates of 3.3% Ag^+^, 95.2% [AuCl_4_]^−^, and 99.8% Fe^3+^. However, after irradiation with UV light, the pore at the opening state exhibited rejection rates of 1.7% Ag^+^, 13.3% [AuCl_4_]^−^, and 99.9% Fe^3+^ (table S4, fig. S36, and Fig. 4D). It is reported that the diameters of hydrated Fe^3+^, [AuCl_4_]^−^, and Ag^+^ are 9.5, 8.0, and 6.8 Å (*47*), respectively (fig. S37). Thus, the hydrated Fe^3+^ and [AuCl_4_]^−^ with a larger molecular size could be mostly rejected by the *trans*-Azo-COF membrane (with a closing-state pore of 0.73 nm), while the hydrated Ag^+^ with a smaller size could pass through the *trans*-Azo-COF membrane easily. Similarly, the hydrated Fe^3+^ with a larger size than the pore width of the *cis*-Azo-COF membrane (with an opening-state pore of 0.93 nm) was still rejected in the feed side, but the hydrated [AuCl_4_]^−^ with a smaller size could permeate through the *cis*-Azo-COF membrane. In other words, the sieving of three metal components could be achieved by using only one Azo-COF membrane with a switchable pore width through UV and vis light irradiation. To prove this concept, a mixed ternary metal aqueous solution was simulated to study the multistage sieving of them (fig. S38). It was found that by using the *trans*-Azo COF membrane, Fe^3+^ and [AuCl_4_]^−^ were excluded into the feed side with rejection rates of 99.9 and 95.8%, respectively, whereas Ag^+^ passed through the pore freely with the rejection rate of only 1.1%. By contrast, the rejection rates of [AuCl_4_]^−^ and Fe^3+^ were 14.8 and 99.8%, respectively, by using the *cis*-Azo COF membrane. As a result, AuCl_4_^−^ was effectively concentrated after the nanofiltration across *trans*/*cis*-Azo-COF membranes with excellent enrichment factors (fig. S39) of Ag^+^ (93.1%), [AuCl_4_]^−^ (94.4%), and Fe^3+^ (99.9%).

### Long-term stability and light-cycling performance of the light-gated COF membrane

The stability of membranes is essential for their industrial application, which may significantly save resources and substantially lower costs. Thus, time-dependent separation performance was investigated to explore the operational stability of the *trans*-Azo-COF and *cis*-Azo-COF membranes in the period of 1440 min (fig. S40). There was no notable decrease in solvent (ethanol and water) flux and solute (CBD and AuCl_4_^−^) rejection, indicating the long-term stability of the membranes. Then, we also conducted the separation of AuCl_4_^−^ by the *trans*-Azo-COF membrane under different pressures (0.5 to 4.5 bar). As can be seen from fig. S41, the value of AuCl_4_^−^ rejection and water permeance remained stable in the studied range of pressure, indicating the stability of the membrane in response to pressure. In addition, combined with the exclusion of feed solute adsorption, this indicates that the molecular rejection and permeance through the Azo-COF membrane followed a size exclusion mechanism that relies only on the size of the pores rather than adsorption.

**Fig. 5.**
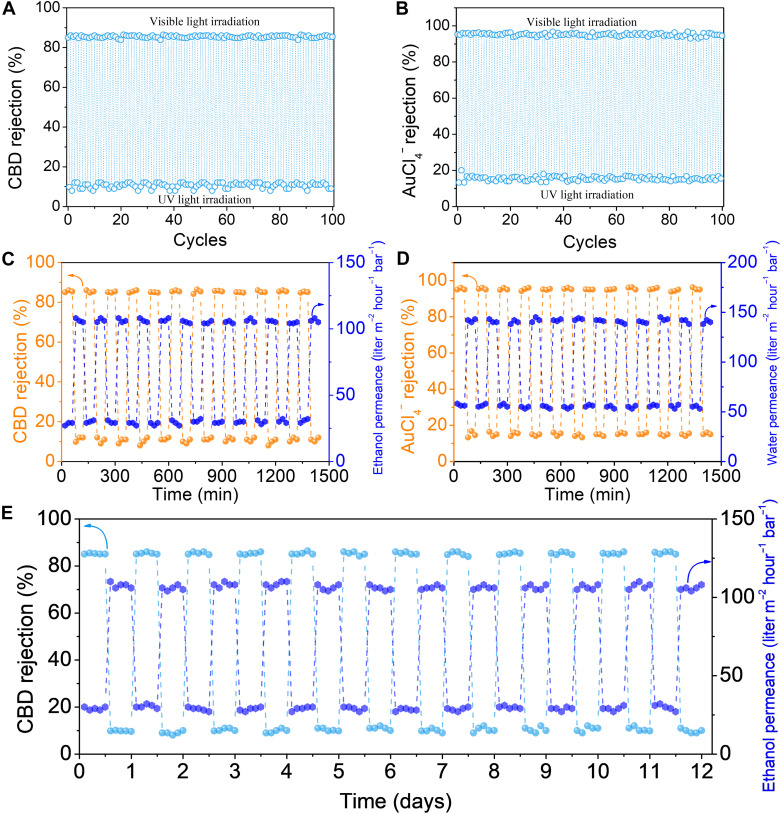
Stability and light-cycling performance of the light-switchable Azo-COF membrane. (**A**) Switched CBD rejection rate of the Azo-COF membrane upon the irradiation of alternative UV and vis light for 100 cycles. (**B**) Switched [AuCl_4_]^−^ rejection rate of the Azo-COF membrane under the irradiation of alternative UV and vis light for 100 cycles. (**C**) CBD rejection and ethanol permeance of the Azo-COF membrane within an extended operational time of 24 hours. (**D**) [AuCl_4_]^−^ rejection and water permeance of the COF membrane within an extended operational time of 24 hours. (**E**) CBD rejection and ethanol permeance of the Azo-COF membrane within the 12-hour operation in the trans state and then 12-hour operation in the cis state for 12 days.

To investigate the reutilization of the Azo-COF membrane in the multistage molecule separation, we performed light-switched solute rejection and solvent permeance under alternative UV and vis light irradiation. It was noted from Fig. 5 (A and B) and fig. S42 that the light-switchable performance was reversible and stable within at least 100 cycles of alternating UV-vis light irradiation. For example, the rejection rate of CBD was switched between 83.3 and 9.8%, and ethanol permeance was switched between 30 and 110 liters m^−2^ hour^−1^ bar^−1^ in CBD separation, while water permeance could be switched between 56 and 142 liters m^−2^ hour^−1^ bar^−1^, and the [AuCl_4_]^−^ rejection rate might be switched between 95.2 and 13.3% in [AuCl_4_]^−^ separation during 100 *trans-cis-trans* cycles. Meanwhile, the change in the rejection rate of limonene, chlorophyll, Ag^+^, and Fe^3+^ was negligible and maintained approximately at ~0.9, ~98.5, ~1.1, and ~99.8%, respectively, in the photocontrolled separation process of ternary solute mixtures by both *trans*- and *cis*-Azo-COF membranes (fig. S43). Furthermore, light-cycling performance testing indicates that the light-responsive stability of the Azo-COF membrane was quite good in 24-hour recycling (Fig. 5, C and D). The enrichment factors were also determined over 100 cycles, and stable values of 96.9 and 94.4% had been observed for CBD and [AuCl_4_]^−^, respectively (fig. S44). After 100 cycles, the PXRD (fig. S45A), TEM (fig. S45B), and FTIR (fig. S45C) characterizations were performed, and no obvious structural changes were detected, confirming the stability of the Azo-COF membrane.

Furthermore, long-term separation experiment was performed to simulate a prolonged operation process, where the membrane was first operated continuously at the trans state for 12 hours and then irradiated by UV light to achieve the cis state, at which another 12 hours was also continuously operated. This two-stage process was continuously operated for 12 days, and ethanol flux and the concentration of each compound were monitored. As can be seen from fig. S46, the membrane exhibited excellent stability during the 12-hour operation at the trans state with the ethanol permeance maintained at ~30 liters m^−2^ hour^−1^ bar^−1^, and the rejection rates of limonene, CBD, and chlorophyll were maintained at ~0.9, 85.1, and 98.3%, respectively. At the cis state, the membrane also showcased excellent stability with an ethanol permeance of ~110 liters m^−2^ hour^−1^ bar^−1^, and the rejection rates of limonene, CBD, and chlorophyll were ~0.8, ~9.8, and ~98.5%, respectively. Importantly, the separation process proceeds with almost unchanged solvent permeance and solute rejection in 12 days (Fig. 5E), together with the high and stable enrichment factors of limonene (85.1%), CBD (95.9%), and chlorophyll (93.5%) (fig. S47). These results provide direct evidence that the membrane’s light-gated separation performance can be sustained over long periods, and the photoswitching functionality remains effective under conditions closer to real-world processes.

## DISCUSSION

Inspired by light-gated channels of the stomata on the leaves, a light-gated COF membrane was designed and fabricated in this work by incorporating azobenzene units onto the wall of nanochannels in an imine-linked COF membrane. For this purpose, the click reaction between alkyne in the COF and azido in the azobenzene derivative was used to increase the grafting content of azobenzene in the COF and to enhance the stability of the Azo-COF membrane. Notably, the as-synthesized Azo-COF membrane exhibited a high grafting rate of azobenzene group of 53.5 wt% (98.5% grafting efficiency) and a highly efficient reversibility of photoisomerization (~100 cycles) resulting from the formation of triazole bonds. It was also found that this light-gated COF membrane could precisely and reversibly switch the pore size between 0.73 and 0.93 nm by alternative irradiation with UV and vis light for highly effective sieving of different sizes of molecules. As a proof of this concept, the Azo-COF membrane was used to sieve limonene, CBD, and chlorophyll in the simulated hemp oil with enrichment factors of 85.1, 95.9, and 93.5%, respectively. The light-gated Azo-COF membrane was also explored to sieve Ag^+^, [AuCl_4_]^−^, and Fe^3+^ in the simulated gold ore with enrichment factors of 93.1, 94.4, and 99.9%, respectively. The light-switched rejection and permeation performance was peculiarly stable for at least 100 cycles under alternating UV-vis light irradiation, and the separation process proceeded with almost unchanged solvent permeance and solute rejection rate in 12 days. Thus, this work provides a concept for constructing light-gated membranes, which could have potential in separations, such as water treatment, separation of high-value medicines, and recovery of precious metals.

In future research, scaling up the fabrication of light-gated COF membranes remains challenging (*48*, *49*). During large-scale synthesis, uneven mass and heat transfer in reactors can cause inconsistent nucleation and growth rates, leading to differences in crystallinity in different regions of the membrane. Moreover, achieving ordered monomer assembly over large areas becomes more difficult, substantially increasing point and grain boundary defects. For interfacial polymerization, maintaining a stable, undisturbed liquid-liquid or gas-liquid interface over extended areas is difficult; minor fluctuations in temperature and concentration or mechanical vibration may result in uneven membrane thickness. For process scale-up, batch processes such as solvothermal synthesis face safety risks, high energy consumption, long reaction time, and poor reproducibility. Although interfacial polymerization allows continuous operation, designing reactors to achieve a large-area, stable, and regenerable interface for monomer replenishment, by-product removal, and continuous transfer of the formed membrane poses engineering challenges. Furthermore, maintaining the structural integrity of large-area COFs without cracking or peeling during application is difficult. Consistency in performance indicators such as permeability and selectivity across batches or regions is also critical for product quality and commercialization.

Promising pathways to address these challenges include the development of continuous flow synthesis technologies, scalable interface polymerization reactors, and integration strategies with mature carriers (*50*, *51*). Continuous flow synthesis is a highly promising strategy. We can explore the synthesis of COF in microchannel reactors, taking advantage of their excellent mass and heat transfer properties to prepare COF nanocrystals, and then coat them for film formation. By means of scalable interface polymerization reactors, for example, designed by the “roll-to-roll” technology, the flexible substrate may continuously pass through the monomer solution pool and the reaction interface to achieve continuous membrane preparation. In addition, integration with mature carriers is the path closest to industrialization, where COF can be used as a selective separation layer, integrated onto inexpensive, sturdy, and already industrially used porous polymer carriers (such as polysulfone and polyvinylidene fluoride) or inorganic carriers (such as alumina ceramics) through in situ growth or coating methods. This can not only reduce the preparation cost but also use the carriers to provide mechanical support, solving the problem of the fragility of COF membranes.

## MATERIALS AND METHODS

### Materials

BPTA (98%) was purchased from Yanshen Technology Co., Ltd. (Jilin, China). *p*-Toluenesulfonic acid (98%), olivetol, 1-methyl-4-(prop-1-en-2-yl)cyclohex-2-enol, chlorophyll, and limonene were acquired from Adamas (Shanghai, China). TAPB (98%), NaN_3_, CuI, *N*,*N*-diisopropylethylamine, HAuCl_4_, chlorophyll, limonene, and *p*-aminoazobenzene were purchased from Aladdin (Shanghai, China). *p*-Aminoazobenzene, NaNO_2_, lithium bis(trifluoromethanesulphonyl)imide, Fe(NO_3_)_3_, and AgNO_3_ were purchased from Macklin (Shanghai, China). All chemicals were used without further purification. A nylon substrate with a diameter of 25 mm and a pore size of 0.2 μm was acquired from Tianjin Jinteng Experimental Equipment Co., Ltd. (Tianjin, China), and used as the support for the freestanding COF membranes.

### Synthesis of Azo

Azo was synthesized according to the previously reported procedures (*52*). For this purpose, *p*-aminoazobenzene (0.197 g, 1 mmol) was added into the solution of *p*-TsOH·H_2_O (1.62 g, 9 mmol) in H_2_O (9 ml). After stirring for 1 min, anhydrous NaNO_2_ (0.621 g, 9 mmol) was added gradually during 5 min. The resulting solution was then stirred for 1 hour until *p*-aminoazobenzene disappeared as monitored by thin-layer chromatography. To the resulting solution, anhydrous NaN_3_ (0.104 g, 1.6 mmol) was added, and immediate emission of N_2_ was observed. After complete reaction, the crude product was filtered, washed with H_2_O, and dried at a vacuum oven overnight. Last, the crude was purified by flash column chromatography (petroleum ether/EtOAc = 20:1, v:v), and the product (denoted as N_3_-azo) was obtained as a brownish-red solid.

### Synthesis of the Azo-COF membrane

The Azo-COF membrane was synthesized via click reaction between the free alkyny moieties in the BPTA-TAPB COF membrane and azido in Azo. Briefly, Azo, CuI, and *N*,*N*-diisopropylethylamine were added into a 10-ml Pyrex tube with 0.5 ml of tetrahydrofuran and 4.5 ml of MeCN as a solvent, and the mixture was subjected to ultrasound treatment for the formation of a homogeneous solution. A piece of the BPTA-TAPB COF membrane was carefully transferred into the tube, which was then kept at room temperature under undisturbed conditions for 24 hours to obtain the Azo-COF membrane after being degassed for three cycles. The membrane was washed thoroughly with DMF and ethanol and then stored in ethanol for further investigations. The synthesis scheme is presented in the Supplementary Materials.

### Membrane nanofiltration experiments

The as-synthesized Azo-COF membrane was transformed onto a nylon substrate to investigate the separation performance (fig. S24). The separation process was performed in a homemade dead-end filtration cell at room temperature with an upstream pressure of 0.5 to 4.5 bar, and continuous stirring was applied to avoid the concentration polarization effect in the separation process. The effective filtration area of the device was 7.854 × 10^−5^ m^−2^, and the volume of the feed side was 150 ml for each solvent or solution. Every time, 5 ml of the filtrate was taken for analytic measurement. At this time, a 5-ml initial feed solution was added to keep the total volume constant. For the separation of HAuCl_4_, AgNO_3_, and Fe(NO_3_)_3_, a 3% HNO_3_ aqueous solution was used as the solvent to avoid any hydrolysis of Au^3+^ and Fe^3+^. At the beginning of the separation measurement, permeation was stabilized for at least 30 min, and then the filtrate was collected for further determination.

## Supplementary Material

20260313-1

## Supplementary Materials

### The PDF file includes:

Experimental section Figs. S1 to S47 Tables S1 to S4 Legend for data S1 References

### Other Supplementary Material for this manuscript includes the following:

Data S1

Erratum published 29 May 2026:
The raw XRD data for both samples in Fig. 2E has been added to the Supplementary Materials as data S1, and the Supplementary Materials PDF has been replaced to include the legend for data S1. The original version of the Supplementary Materials PDF is available here:
