# Supplementary material for: Highly efficient light-gated COF membrane for precise multistage molecular separation
Source: Sci Adv. 2026 Mar 13;12(11):eadz1929. doi: 10.1126/sciadv.adz1929 (PMC12985721; doi:10.1126/sciadv.adz1929)
Supplement: Supplementary file 2 — Experimental section Figs. S1 to S47 Tables S1 to S4 Legend for data S1 References [file sciadv.adz1929_sm.v2.pdf]

ERRATUM PUBLISHED 5 JUNE 2026; SEE BELOW

Science Advances
AAAS

Supplementary Materials for

Highly efficient light-gated COF membrane for precise multistage molecular separation

Liyong Zhai *et al.*

Corresponding author: Shuaiqi Gao, gaoshuaiqi@htu.edu.cn; Zhiyong Li, yli@htu.edu.cn; Jianji Wang, jwang@htu.edu.cn

Sci. Adv. **12**, eadz1929 (2026)
DOI: 10.1126/sciadv.adz1929

The PDF file includes:

Experimental section
Figs. S1 to S47
Tables S1 to S4
Legend for data S1
References

Other Supplementary Material for this manuscript includes the following:

Data S1

Erratum (5 June 2026): The original Supplementary Materials did not include the raw data for Fig. 2. The raw XRD data for both samples in Fig. 2E has been added to the Supplementary Materials files as data S1. The Supplementary Materials PDF has been replaced to include the legend for data S1. The authors' results and conclusions are not affected by this correction.

1. Experimental

1.1 Sample characterizations

Powder X-ray diffraction (PXRD) patterns were collected using a D8 Advance diffractometer of Brukeraxs with Cu Kα radiation (λ = 0.15418 nm) at 40 kV and 30 mA, and the scanning rate was 0.01°s^{-1}. The surface morphology and cross-sectional structure of the membranes were analyzed using a Hitachi SU8010 microscope operated at an accelerating voltage of 10.0 kV. A high-resolution TEM microscope (300 kV, FEI Titan CT, USA) was used to analyze membrane structure. Fourier transform infrared (FT-IR) spectra were collected on a Spectrum 400 spectrometer (Perkin-Elmer) to analyze chemical structures of the azo-COF membranes and their monomers. X-ray photoelectron spectra (XPS) were determined by a Thermo Scientific K-Alpha electron energy spectrometer using Al Kα (1486.6 eV) radiation as the X-ray excitation source. X-ray photoelectron spectroscopy (XPS) depth profile analysis was conducted using argon-ion etching with a sputter rate of 0.2 nm s^{-1} and the total etching time was 30 min. Solid-state ^{13}C cross-polarization magic-angle-spinning nuclear magnetic resonance (^{13}C CP-MAS NMR) spectroscopy was also used to probe chemical structures of the COF and azo-COF membranes. UV-vis spectra of the membranes and the monomers were determined under UV 365-nm irradiation/vis-light irradiation by an UV-vis spectrometer (Evolution 220, Thermo Fisher Scientific, USA). Nitrogen adsorption-desorption experiment performed at 77 K was used for the estimation of membrane porosity via surface characterization analyzer (ASiQwin Quantachrome instrument). The samples were evacuated at 120 °C for 12 hours before the measurement. The thermal stability of monomers and membranes was evaluated by TGA with a TA Instrument (TGA-Q50) from 25 to 800 ℃ at a heat ramp of 10 °C/min. The contact angles were determined by a contact angle tester (Biolin Theta). The content of elements including C, H, and N was measured by an elemental analyzer (Thermo Fisher Scientific Inc.). The concentration of the metal ions was determined by inductively coupled plasma optical emission spectroscopy (ICP-OES) through an Agilent 5800 instrument. Before the ICP-OES analysis, the solution was diluted to meet the standard curves used for quantification. Agilent 12605042LOCK series HPLC with SuperC18 column (250 × 4.6 mm) was used to analyze the concentrations of CBD and limonene. The HPLC mobile phases were prepared by dissolving 0.1% formic acid in water and acetonitrile, respectively.

1.2 Synthesis of 1-decyl-3-methylimidazolium bis ((trifluoromethane) sulfonyl) imide

1-Decyl-3-methylimidazolium bis((trifluoromethane)sulfonyl)imide ionic liquid ([C_{10}Mim][Tf_2N]) was prepared and purified as described in the literature (*53*). Briefly, aqueous lithium bis(trifluoromethanesulphonyl)imide (28.71 g, 0.1 mol) was added drop-wise to a solution of 1-methyl-3-butylimidazolium bromide (15.68 g, 0.098 mol) in dichloromethane and stirred at room temperature overnight. The organic layer was extracted, washed with distilled water and brine, dried over anhydrous $MgSO_4$. Finally, the residual solvent was removed under reduced pressure to yield light yellow liquid as [C_{10}Mim][Tf_2N].

1.3 Synthesis of BPTA-TAPB COF membrane

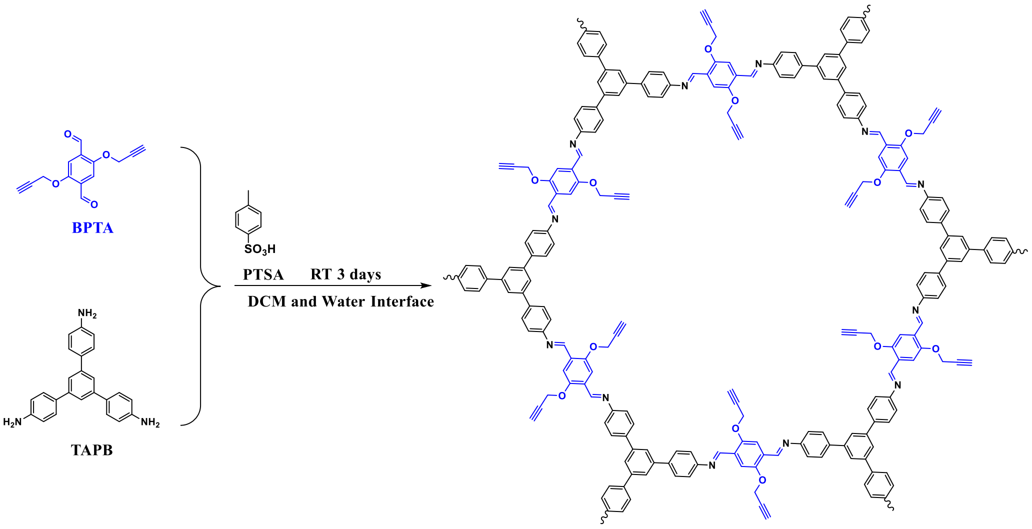

The BPTA-TAPB COF membrane was synthesized via Schiff base reaction between 2,5-bis(2-propynyloxy) terephthalaldehyde (BPTA) and 1,3,5-tris (4-aminophenyl)benzene (TAPB) at IL-water interface with p-toluenesulfonic acid (PTSA) as the catalyst. Here, the hydrophobic ionic liquid was used with water to form IL-water interface. For this purpose, 25 mg of BPTA was dissolved in 35 mL of [C_{10}Mim][Tf_2N] to give a 3 mM solution of aldehyde (denoted as solution A). Then, 3 mM aqueous

solution of TAPB was prepared by dissolving 53 mg of TAPB in 50 mL of water with 86 mg of PTSA as catalyst (denoted as solution B). After that, 3 mL of solution A was transferred to a 10 mL vial as bottom layer, and 3 mL of water was added on the top of the IL layer to form a spacer layer of water, and 3 mL of solution B was added slowly to the top of the spacer layer in 2 min as the top layer. The above system was kept at room temperature under undisturbed conditions for 72 h to obtain the BPTA-TAPB COF membrane. The membrane formed at the interface was collected and washed thoroughly with dichloromethane and ethanol in turn, which was then put into ethanol for further investigations.

1.4 Synthesis of cannabidiol oil

The cannabidiol oil (CBD) was synthesized according to previously reported procedures (*37*). A solution of olivetol (0.40 g, 2.2 mol) and p-TsOH·H_2O (40 mg, 0.21 mmol) in toluene (28 mL) was refluxed for 2.5 h. Cooled to room temperature and added 1-methyl-4-(prop-1-en-2-yl)cyclohex-2-enol (0.47 g, 3.1 mmol). The mixture was stirred for 0.5 h to obtain the crude product, which was then purified by flash chromatography to afford the target compound CBD.

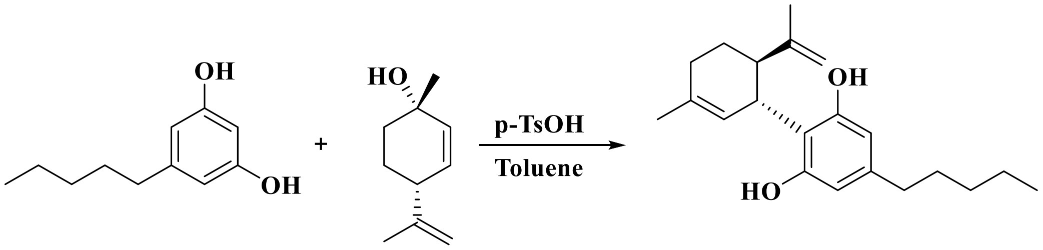

1.5 Membrane nanofiltration and solvent permeation experiments

The molecule rejection rate (R) was calculated by the following equation (*54*)：

$$R=(1-C_p/C_f)\times 100\% \quad (1)$$

where C_p and C_f are the concentration of permeate and feed solutions, respectively, which was measured by UV-vis spectroscopy or inductively coupled plasma optical emission spectroscopy (ICP-OES). To exclude the effect of solute adsorption, both permeance and rejection were collected when a steady permeate flux was achieved. At least three parallel membrane samples were tested to obtain reliable solvent permeance and solute rejection data.

The solvent permeance of the COF membranes was calculated by the following equation (*11*) :

$$P=V/(A\times\Delta t\times p) \qquad (2)$$

where V(L) is the volume of the solvent permeate, A (m^{-2}) is the effective filtration area, Δt is the permeate time (h) and p is the applied pressure (bar) at the permeance process.

The analysis of the enrichment factor reported in the previous research (*55*) was slightly modified and then applied to this study. The enrichment factors of CBD and $[AuCl_4]^-$ were determined under continuous filtration conditions, where 150 mL of the feed solution was used for the nanofiltration by *cis*-Azo-COF membrane and 50 mL of filtrate was collected to be the new feed solution for the nanofiltration by *trans*-Azo-COF membrane. After collecting 10 mL filtrate, the concentrations of CBD and $[AuCl_4]^-$ in residual feed solutions were determined for one cycle. The enrichment factor (EF) was calculated using the following equation (*56*):

$$EF=C_{Goal}/C_{Total} \qquad (3)$$

where C_{Goal} represents the concentration of target solute in the final solution, and C_{Total} stands for the concentration of all solutes in the final solution.

1.6 Molecular weight cutoff and pore size distribution of the Azo-COF membrane

Water based molecular weight cutoff (MWCO) and pore size distribution of the membrane was determined by the nanofiltration of neutral PEG with average molecular weight of 200, 400, 600, 800, 1000 Da at the concentration of 0.5 g L^{-1} and the pressure of 1.0 bar. Ethanol based molecular weight cutoff and pore size distribution of the membrane was determined by the nanofiltration of five standard dyes with known molecular weight (p-Nitrophenol (139 Da), methyl orange (327 Da), Rhodamine B (479 Da), Congo red (697 Da) and Coomassie brilliant blue R250 (827 Da) at the concentration of 25 mg L^{-1} and the pressure of 1.0 bar. The MWCO value of the membranes was defined as the molecular weight at which the rejection equals 90%. The filtration tests were operated in the cross-flow filtration system and the concentrations of PEG solutions were analyzed by UV-vis spectroscopy. The Stokes diameter of PEG was calculated from the following equation: (*57, 58*)

$$d_s=33.46\times 10^{-12}\times M^{0.557} \qquad (4)$$

where d_s and M represent Stokes diameter and molecular weight of PEG solute. The probability density function curve of the membrane pore size was calculated by the following equation: (*30*)

$$\frac{dR(r_p)}{dr_p} = \frac{1}{r_p \ln \sigma_p \sqrt{2\pi}} exp\left[-\frac{(lnd_p - ln\mu_p)^2}{2(ln\sigma_p)^2}\right] \qquad (5)$$

where μ_p is the average effective pore size determined at the solute rejection R = 50%, and σ_p is the geometric standard deviation, which is defined by dividing the rejection ratio at R = 84.13% and R = 50%.

2. Figures S1 to S47

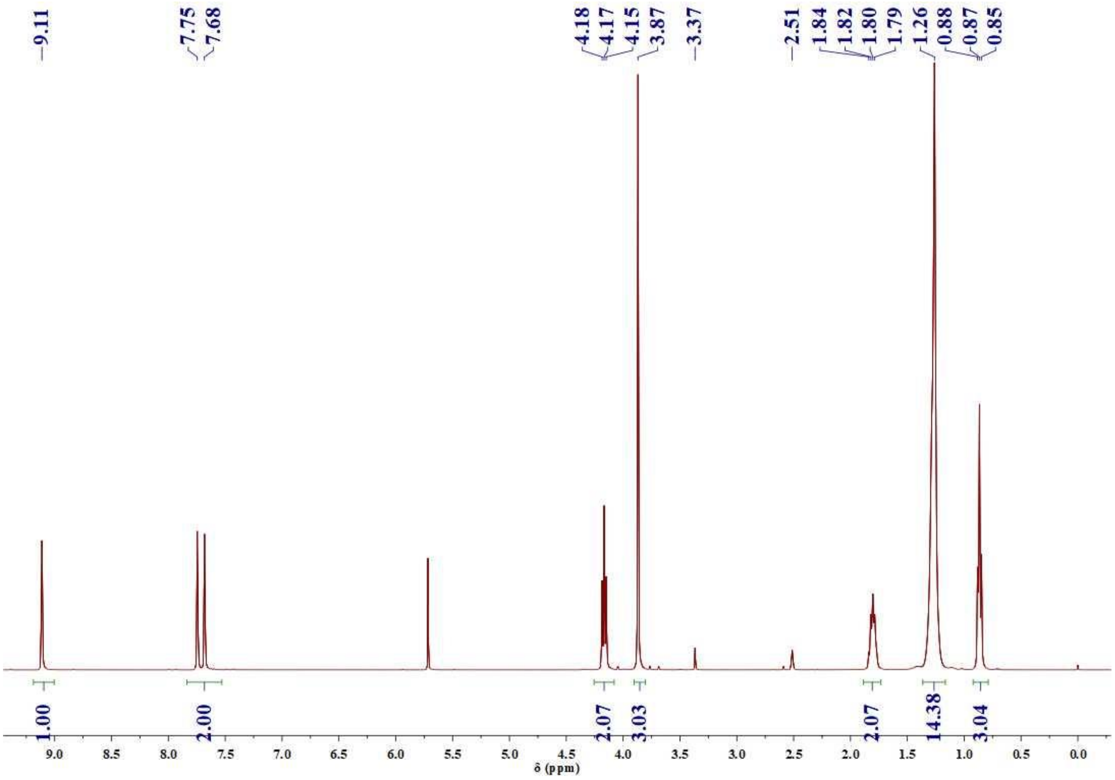

Fig. S1. ^{1}H NMR spectrum of 1-decyl-3-methylimidazoliumbis-((trifluoromethane)-sulfonyl) imide ([C_{10}Mim][Tf_2N]).

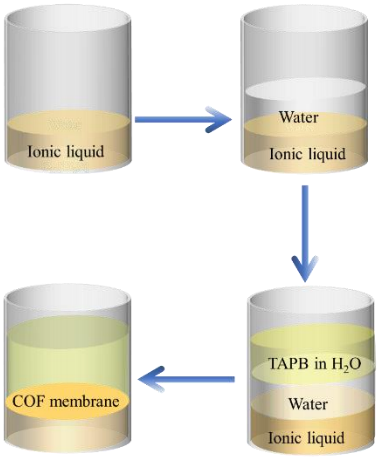

Fig. S2. Fabrication schematic of the COF membrane via interfacial reaction. Note: aldehyde was in ionic liquid phase and amine/PTSA (catalyst) was in water phase.

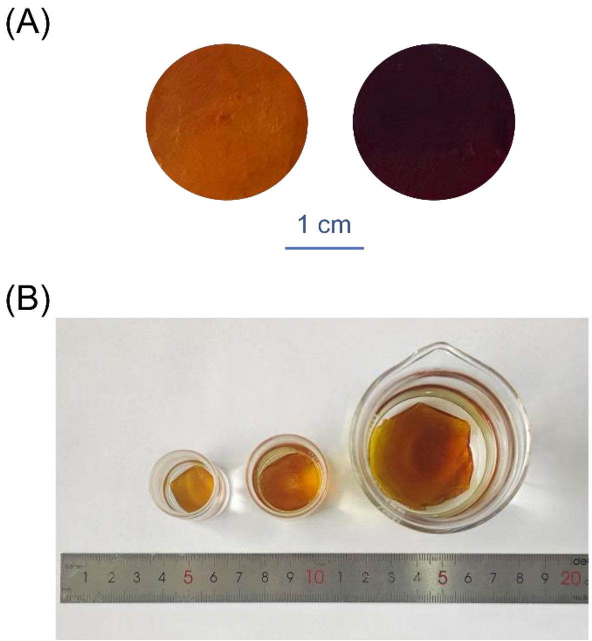

Fig. S3. (A) The photograph of freestanding BPTA-TAPB COF (left) and Azo-COF membranes (right). (B) Digital images of the 25 mm, 30 mm and 50 mm COF membranes.

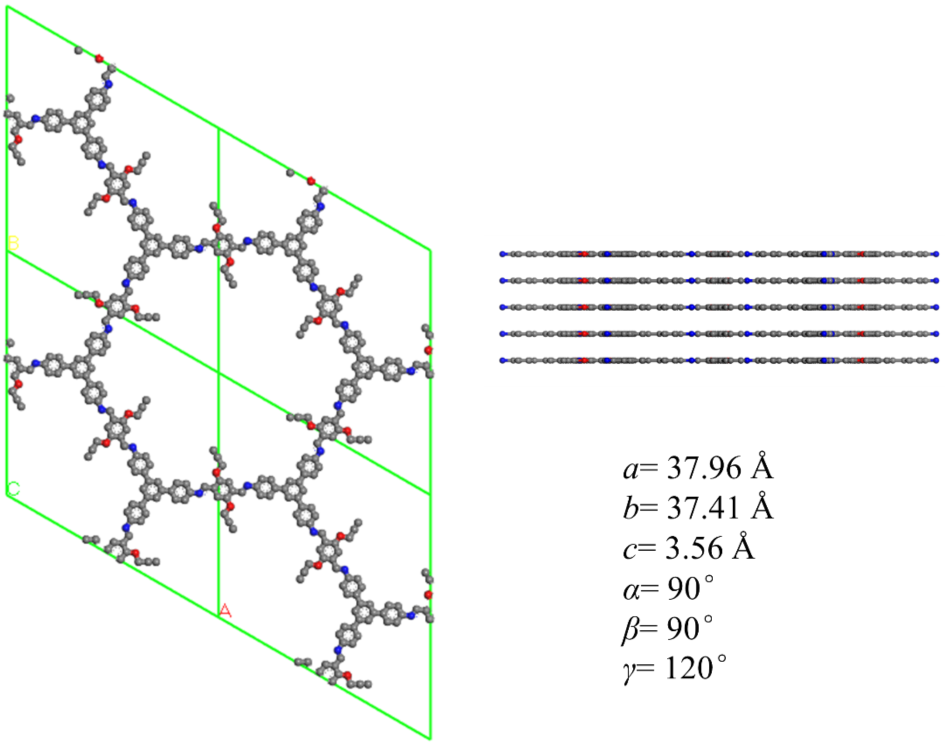

Fig. S4. The simulated structure of BPTA-TAPB COF membrane in x-y and z side views.

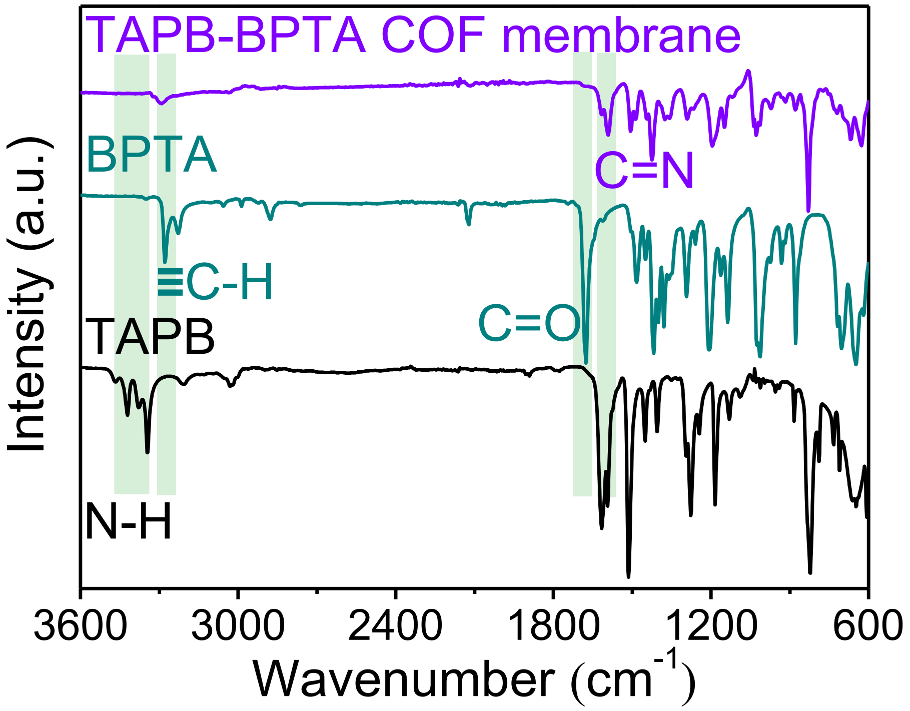

Fig. S5. FT-IR spectra of the monomers and BPTA-TAPB COF membrane.

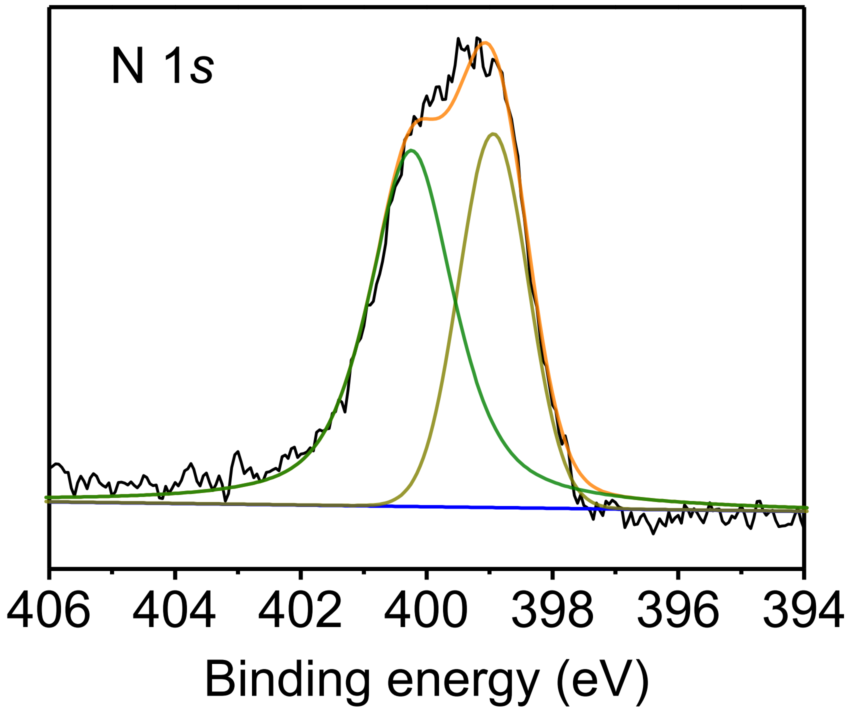

Fig. S6. XPS spectrum of the BPTA-TAPB COF membrane.

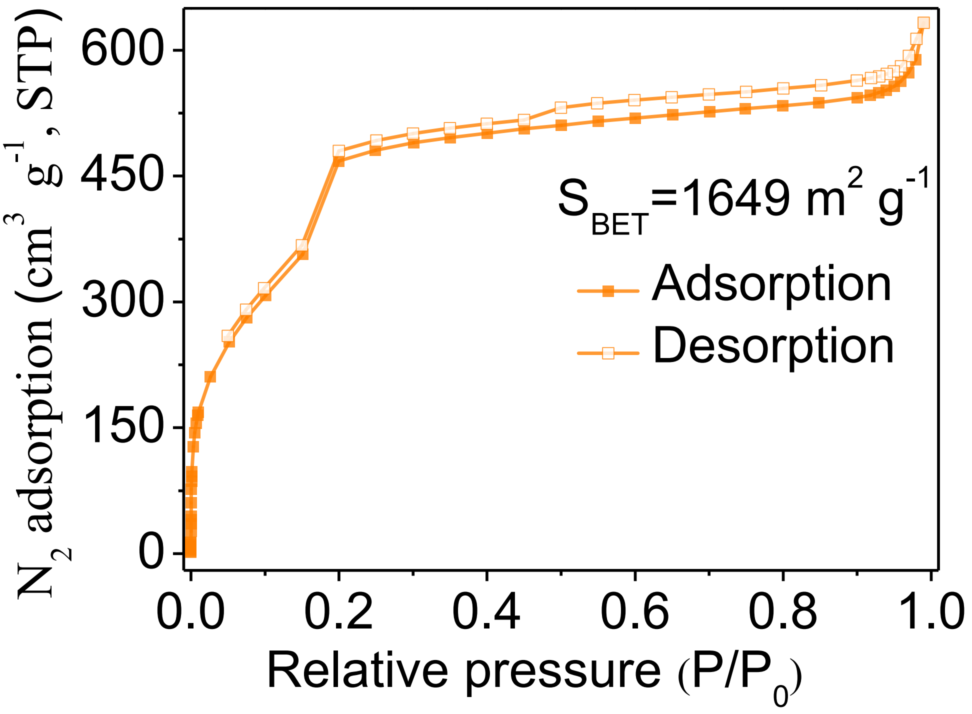

Fig. S7. N_2 adsorption-desorption isotherm of BPTA-TAPB COF membrane.

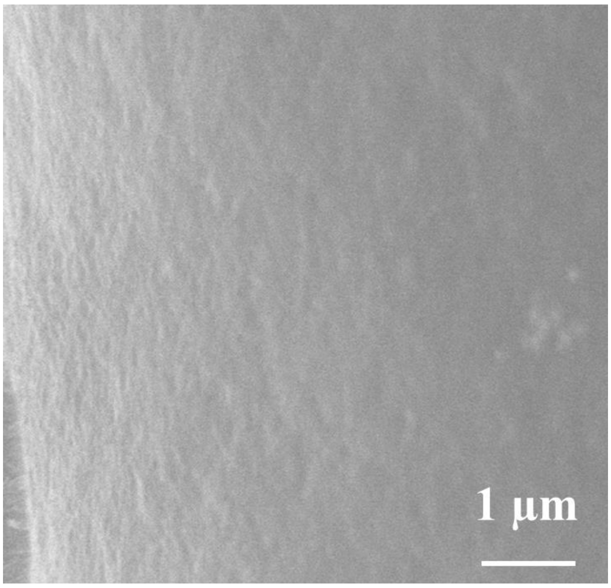

Fig. S8. SEM image of the BPTA-TAPB COF membrane.

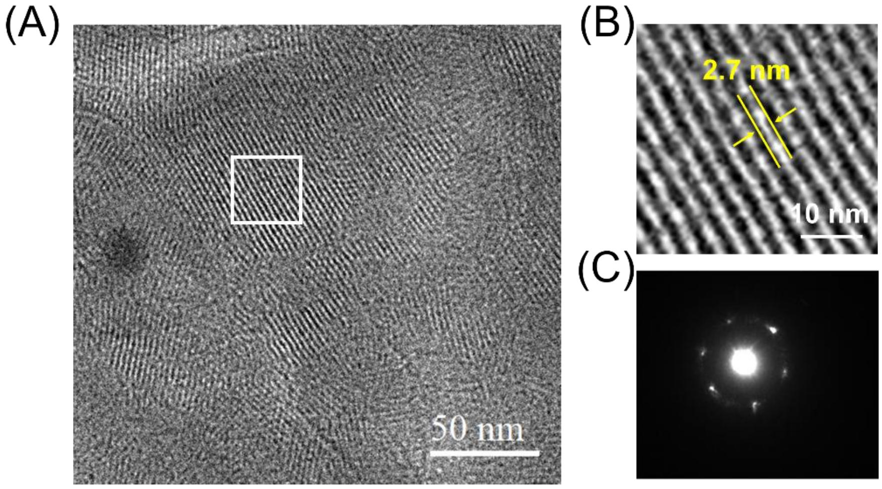

Fig. S9. HRTEM images of the BPTA-TAPB COF membrane.

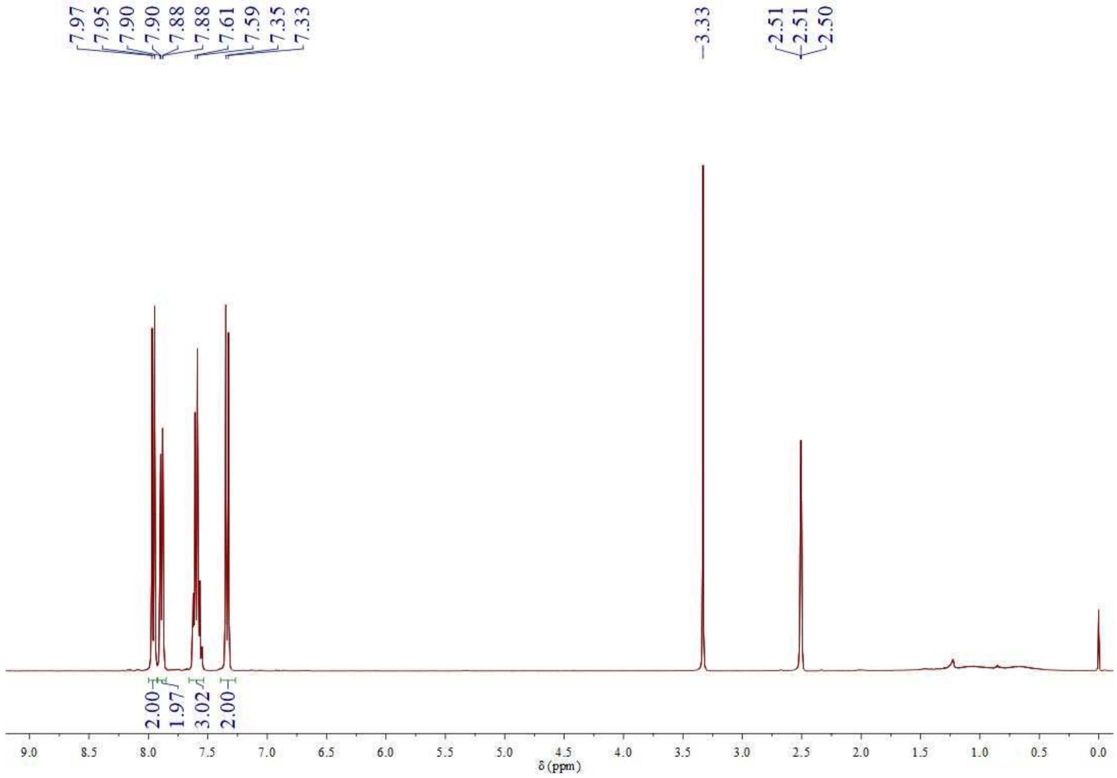

Fig. S10. ^{1}H NMR spectrum of 4-azidophenylazobenzene (N_3-Azo).

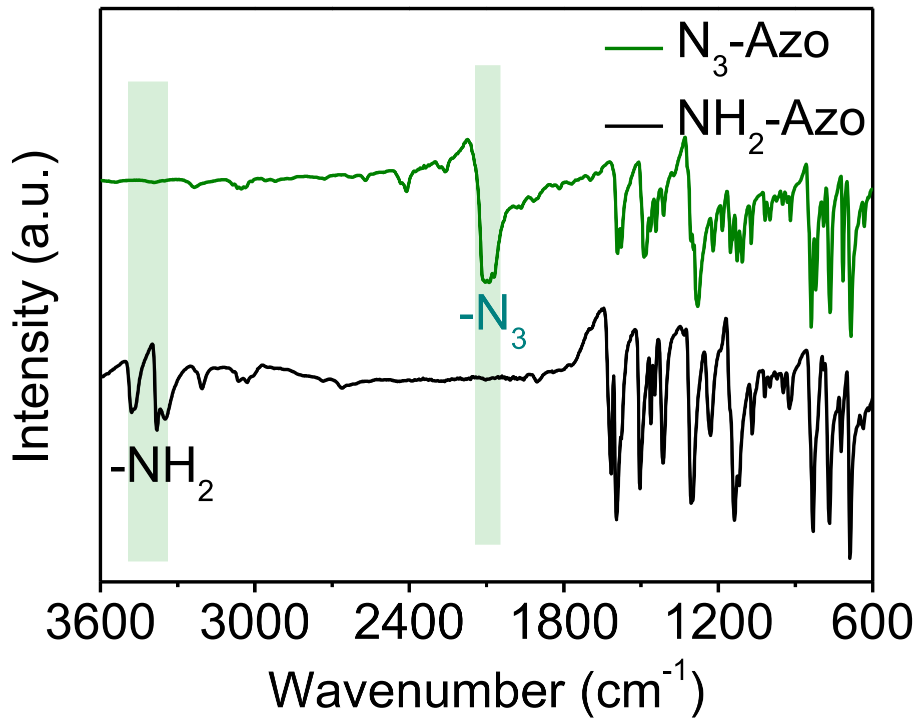

Fig. S11. FT-IR spectra of 4-aminoazobenzene (NH_2-Azo) and 4-azidophenyl-azobenzene.

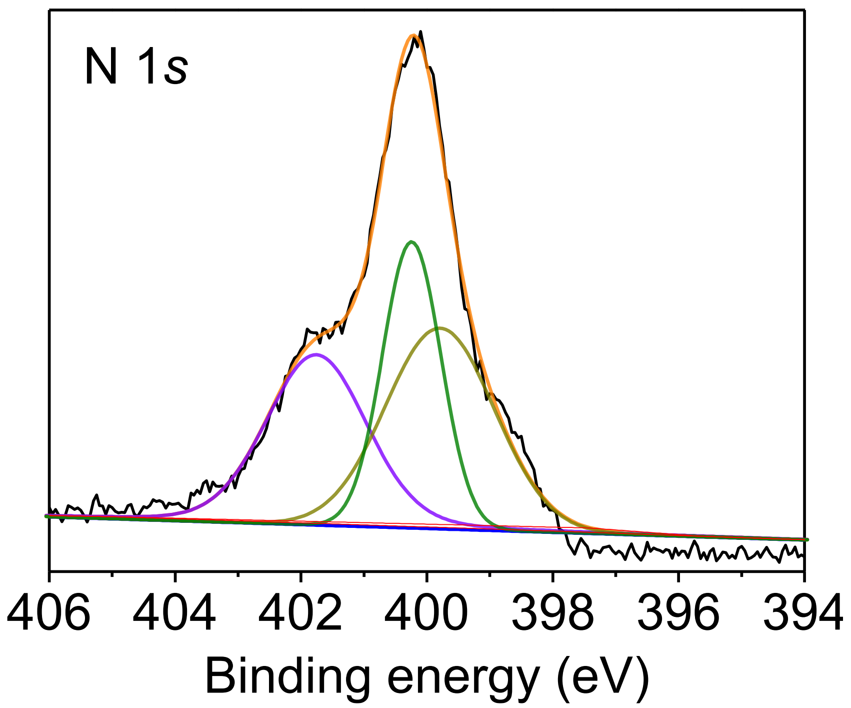

Fig. S12. XPS spectrum of the Azo-COF membrane.

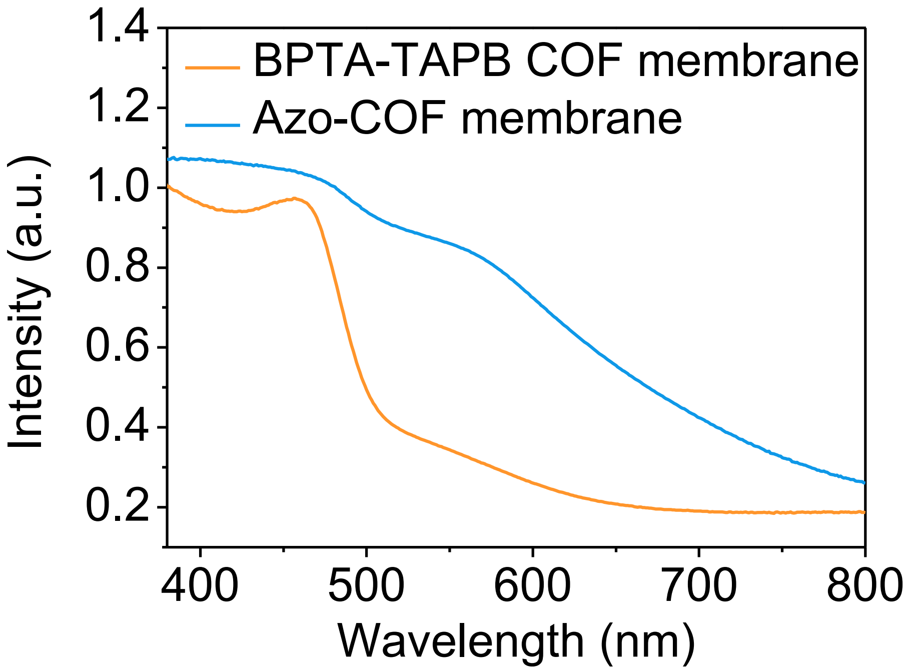

Fig. S13. DRS spectra of the COF membrane before and after Azo grafting.

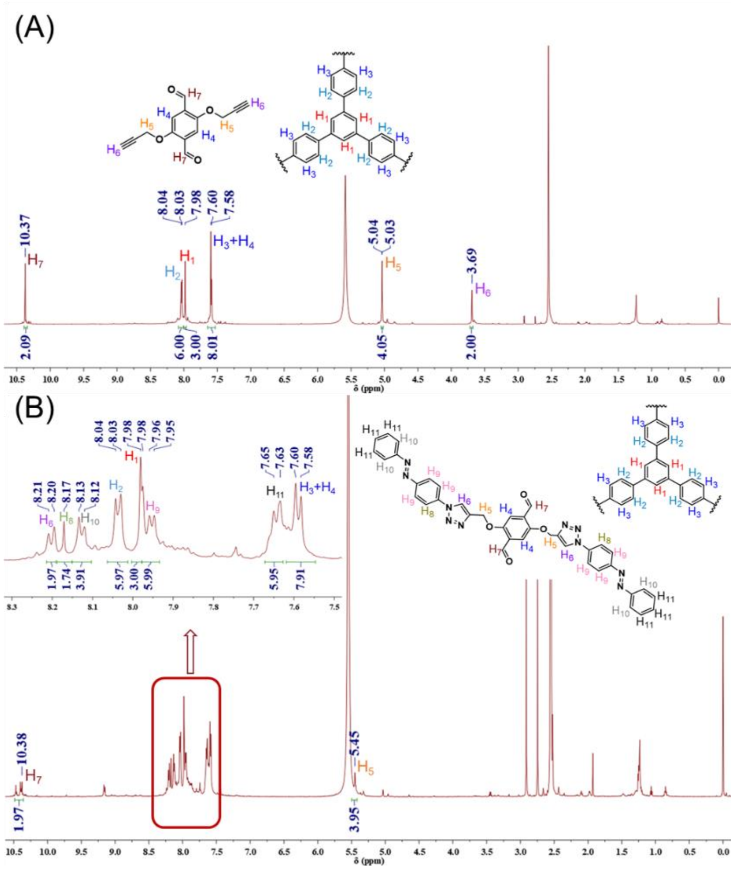

Fig. S14. ^{1}H NMR spectra of the digested (A) pristine BPTA-TAPB COF membrane and (B) Azo-COF membrane in DMSO-d_6. Note: The membranes are digested by DCl (20 wt.% in D_2O) at 85 ℃ for 5 h to release the molecular building blocks (amine and aldehyde monomers).

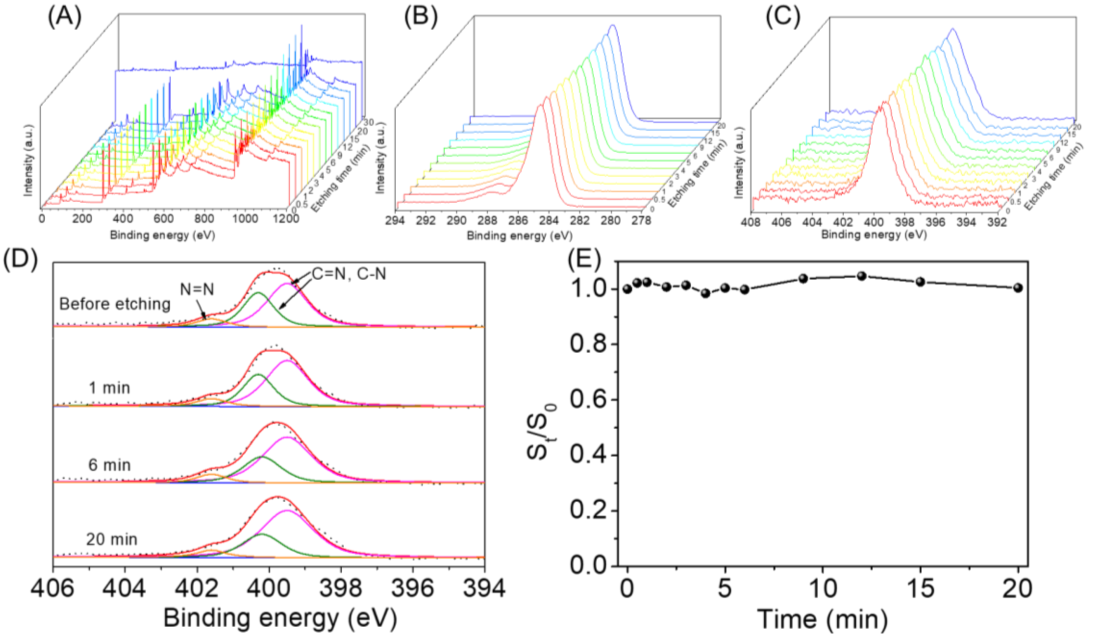

Fig. S15. (A) High-resolution XPS spectra, (B) C *1s* and (C) N *1s* depth profiles of Azo-COF membrane. (D) Curves of the variation of N *1s* at different etching time. (E) Curves of the variation in the ratio between the N=N peak areas (S_t) at different etching times and (S_0) for the unetched membrane surfaces.

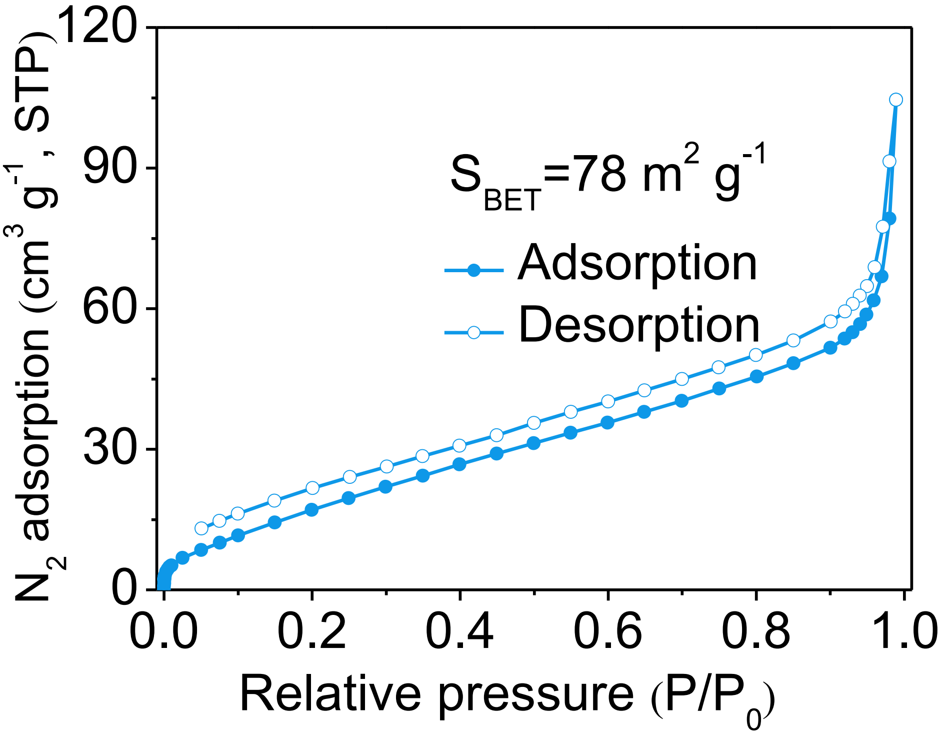

Fig. S16. N_2 adsorption-desorption isotherm of Azo-COF membrane.

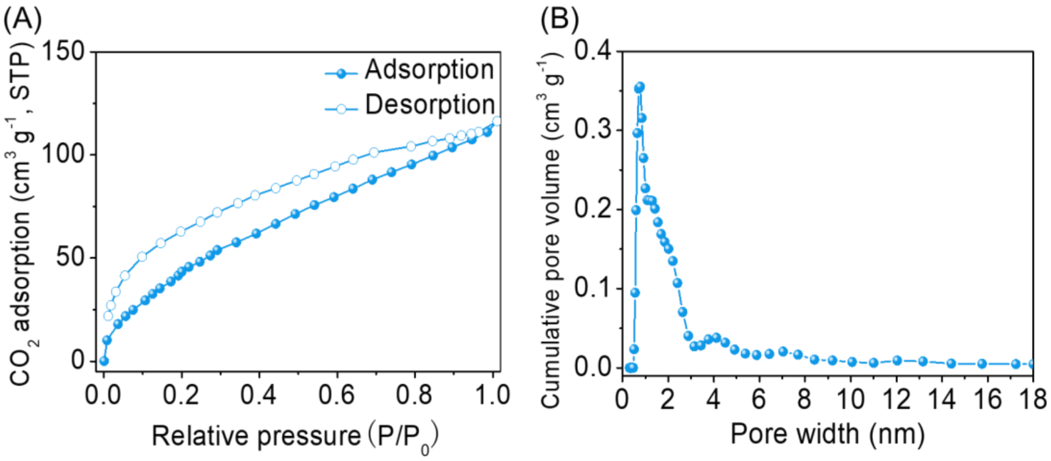

Fig. S17. CO_2 adsorption isotherms and pore size distribution of Azo-COF membrane at 195 K.

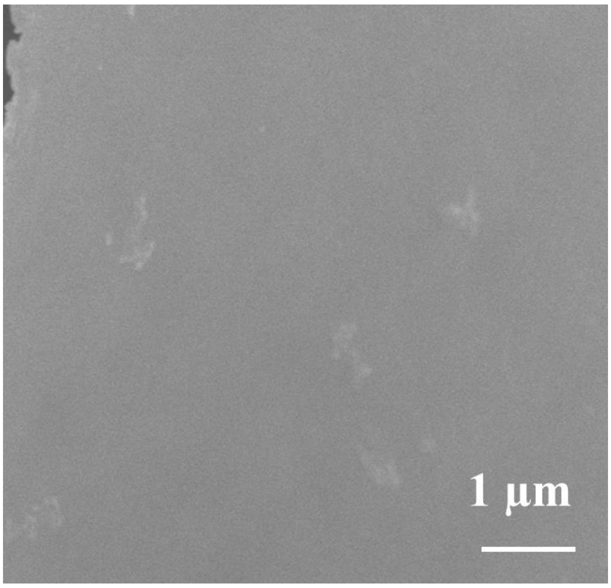

Fig. S18. SEM image of the Azo-COF membrane.

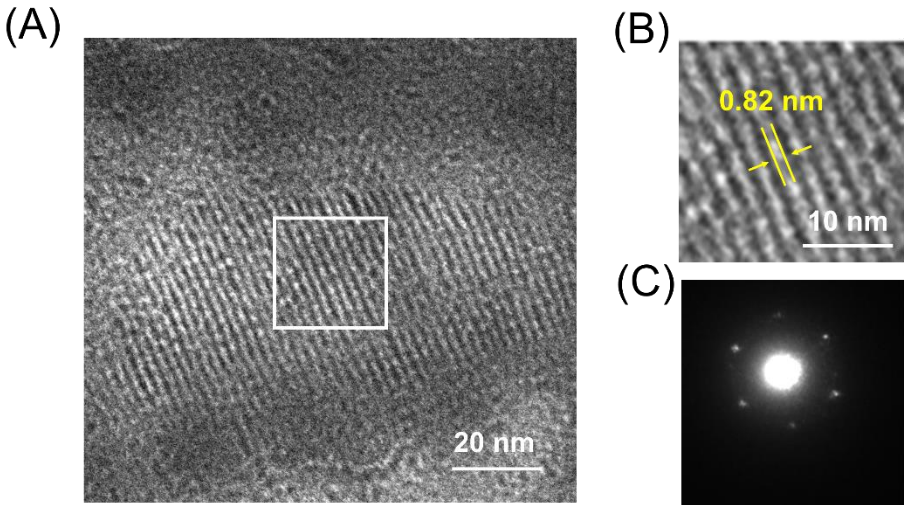

Fig. S19. HRTEM images of the Azo-COF membrane.

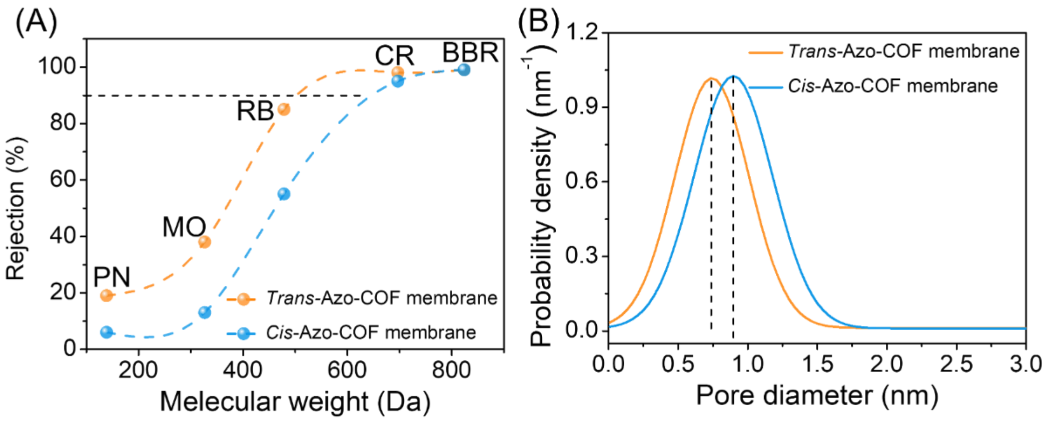

Fig. S20. (A) Rejection curves of the *trans/cis*-Azo COF membranes for standard dyes. (B) Pore size distribution of the *trans/cis*-Azo COF membranes.

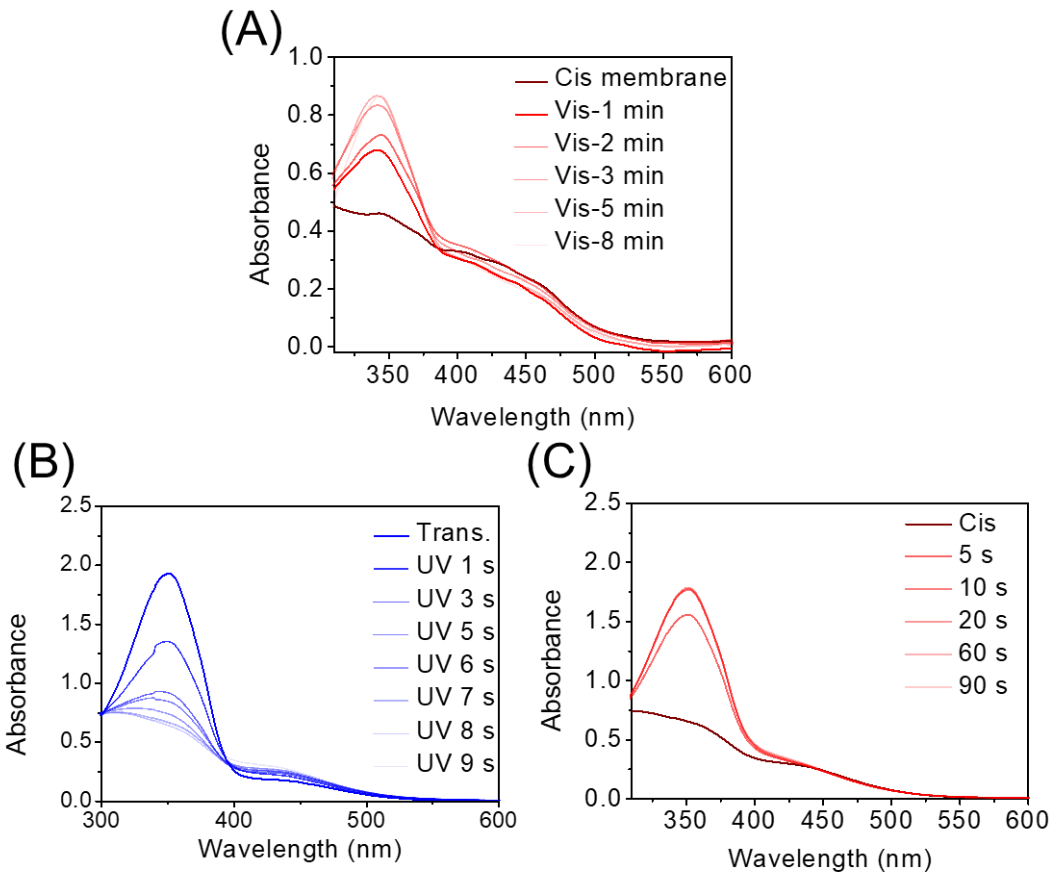

Fig. S21. The photoisomerization of (A) Azo-COF membrane. The photoisomerization of N_3-Azo monomer under (B) UV and (C) visible light irradiation.

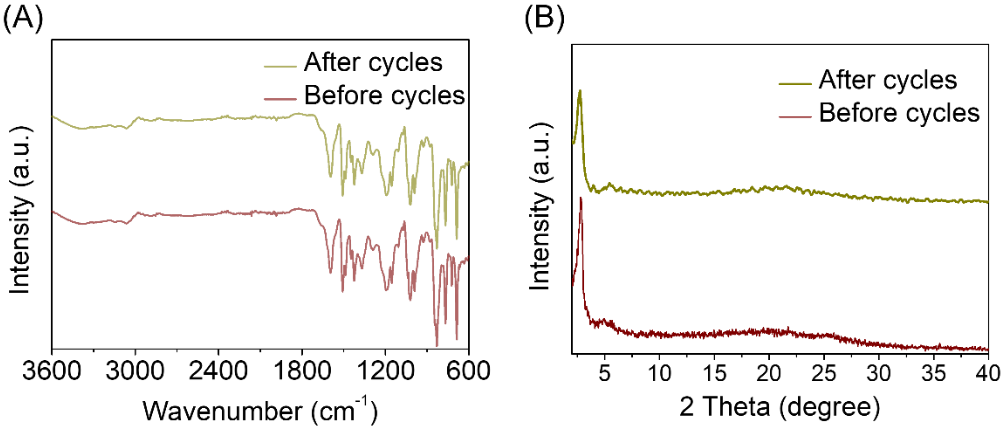

Figure S22. (A) The FT-IR spectra and (B) PXRD patterns of Azo-COF membrane after 100 cycles.

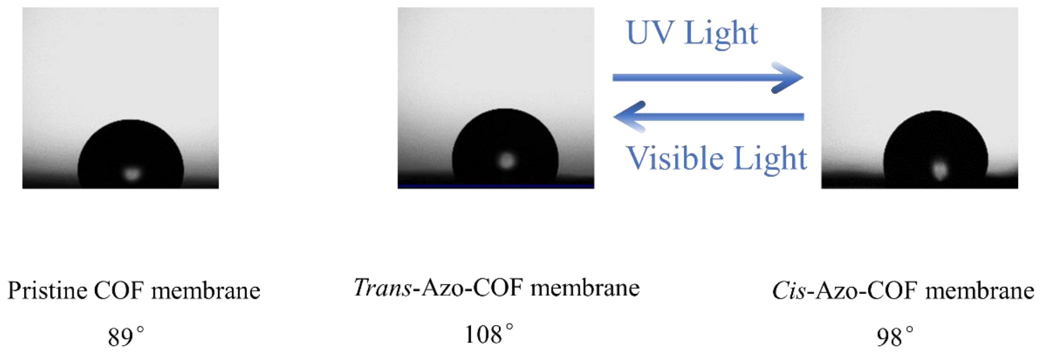

Fig. S23. Contact angle of the pristine COF membrane and Azo-COF membrane.

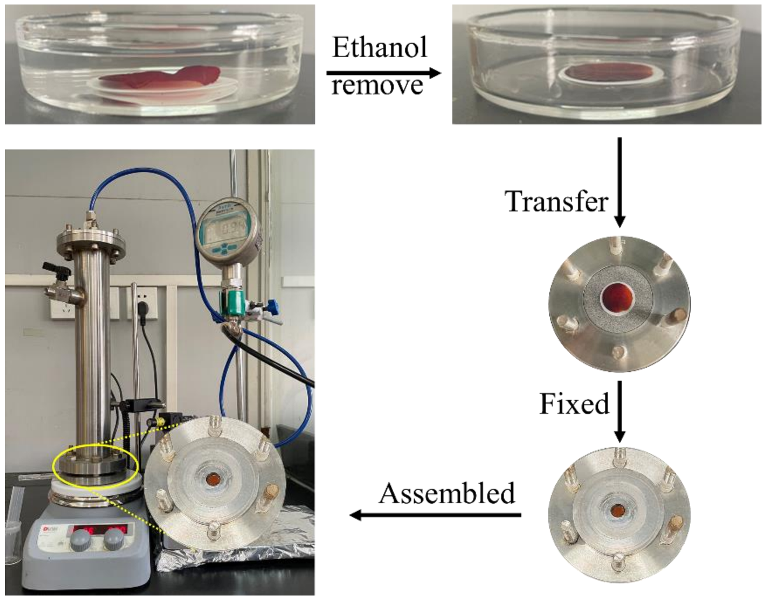

Fig. S24. The schematic of COF membrane transformed onto a nylon substrate and the home-made dead-end filtration cell. The obtained freestanding COF membrane was simply transformed onto a nylon substrate in ethanol without any other treatment.

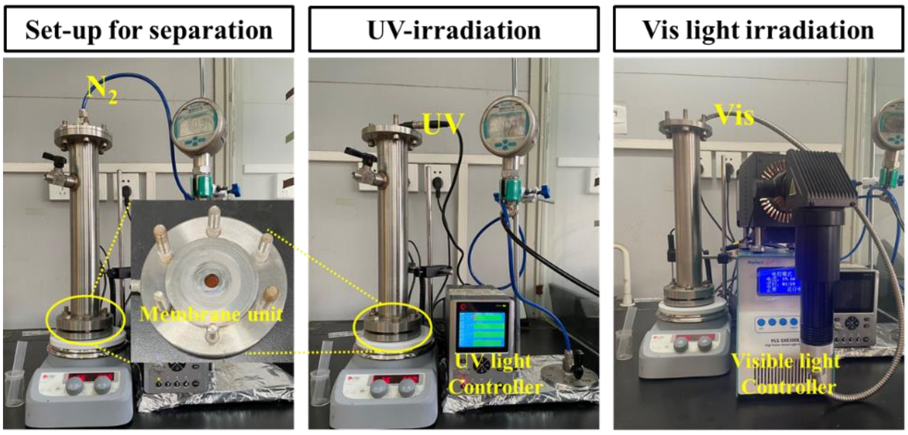

Fig. S25. The set-up used for the light-controlled separation process. The light was provided by the UV light controller and the visible light controller, respectively. During light irradiation process, UV and visible light was only applied for the COF membrane but not nylon support.

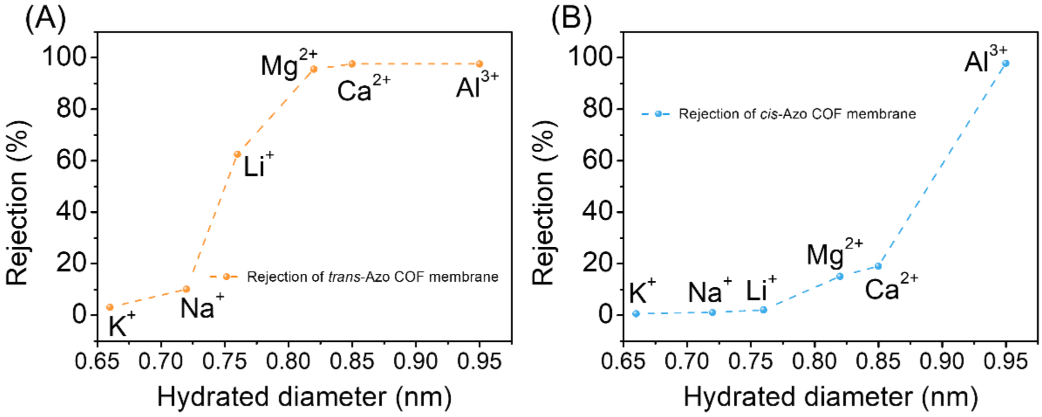

Fig. S26. Rejection of K^+, Na^+, Li^+, Mg^{2+}, Ca^{2+} and Al^{3+} across (A) *trans*-Azo COF membrane and (B) *cis*-Azo COF membrane.

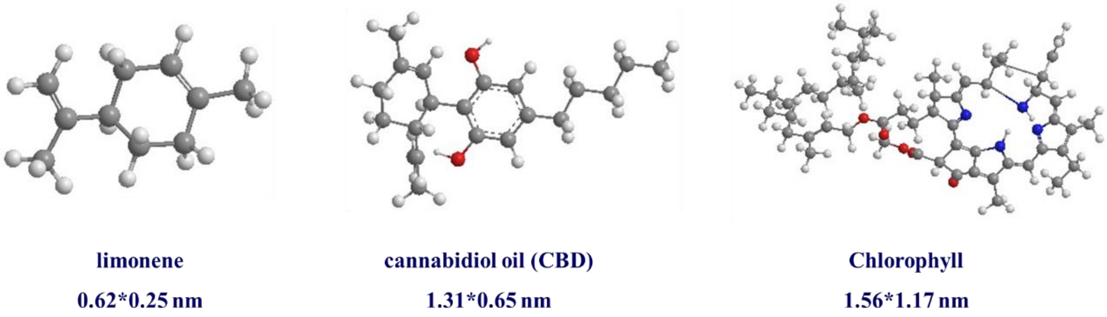

Fig. S27. The chemical structure and molecular size of CBD, limonene and chlorophyll.

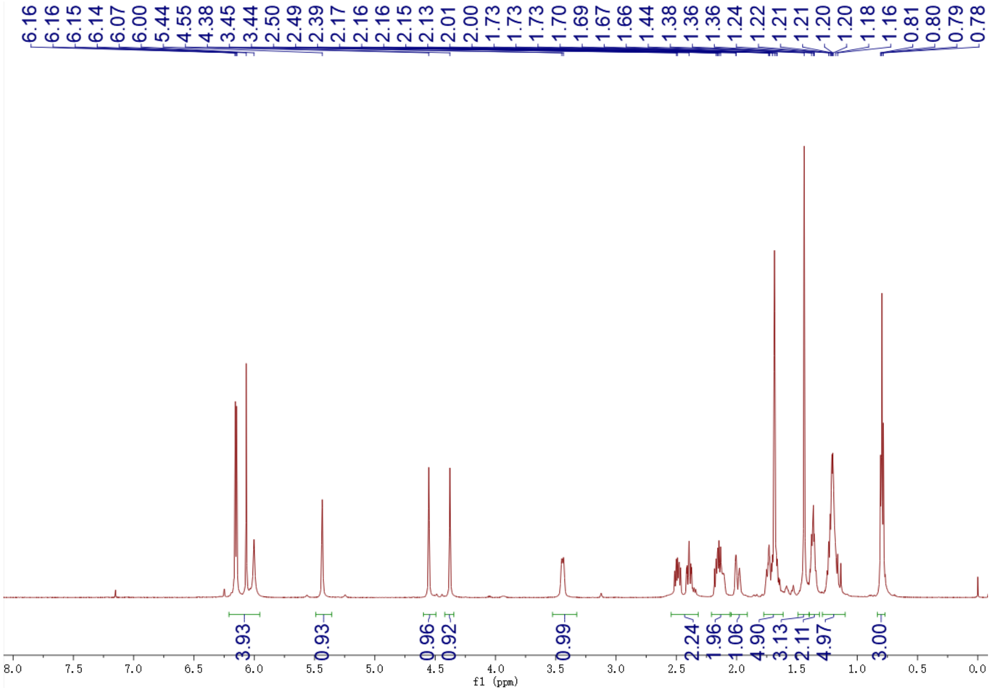

Fig. S28. ^{1}H NMR spectrum of cannabidiol oil (CBD).

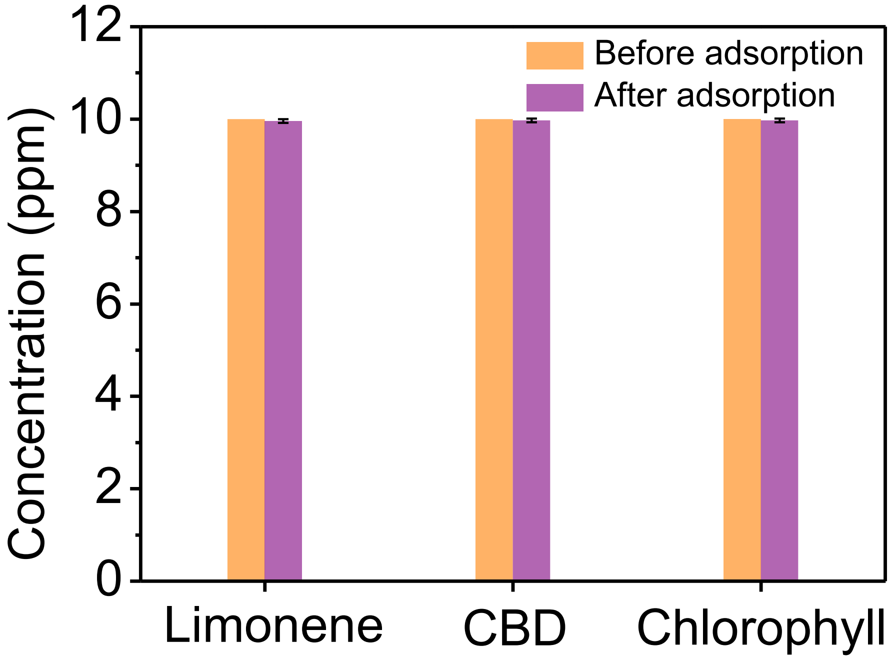

Fig. S29. The concentrations of CBD and its competition molecules in feed solution before and after the membrane was immersed in the solution for 24 h.

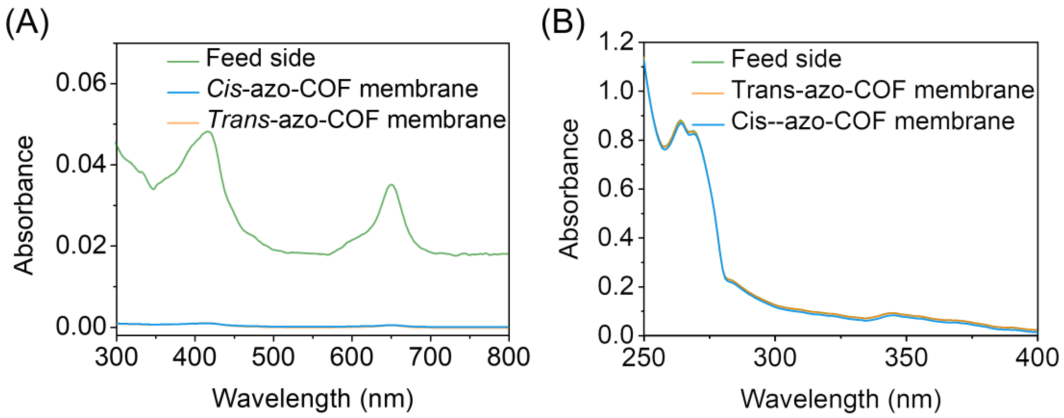

Fig. S30. UV-vis spectra of (A) chlorophyll and (B) limonene at the permeate side through *trans/cis*-Azo-COF membrane.

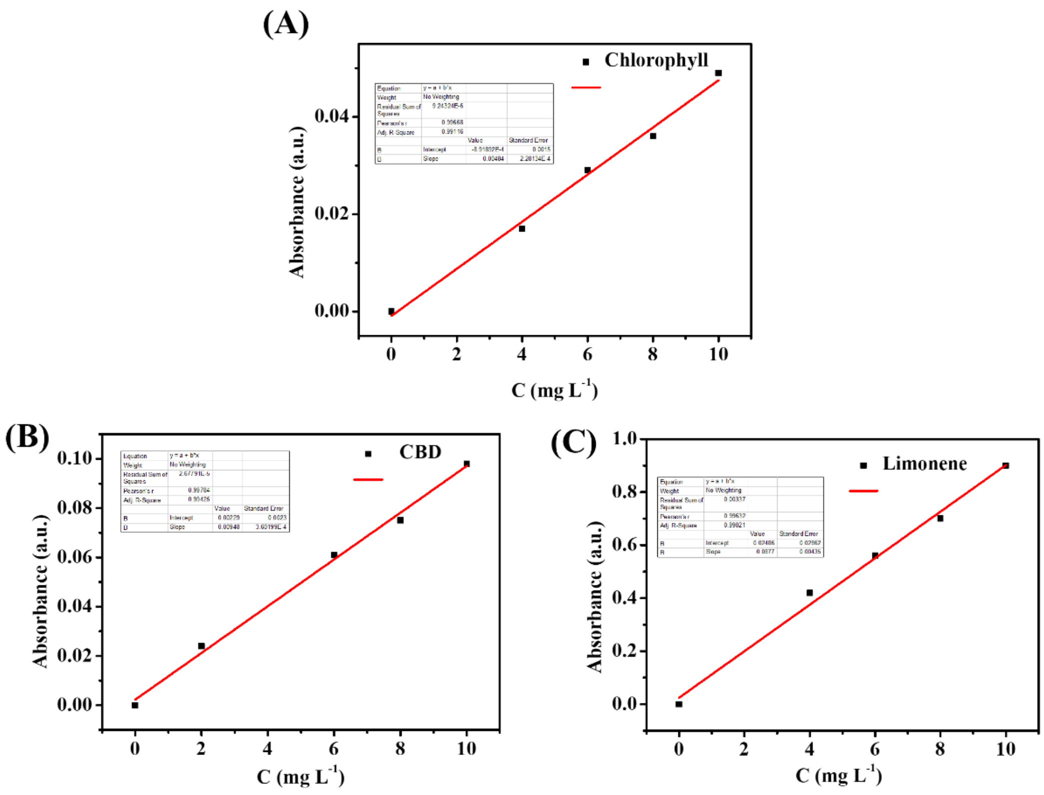

Fig. S31. Standard curves of CBD and its competition molecules in ethanol from UV-vis spectral measurements.

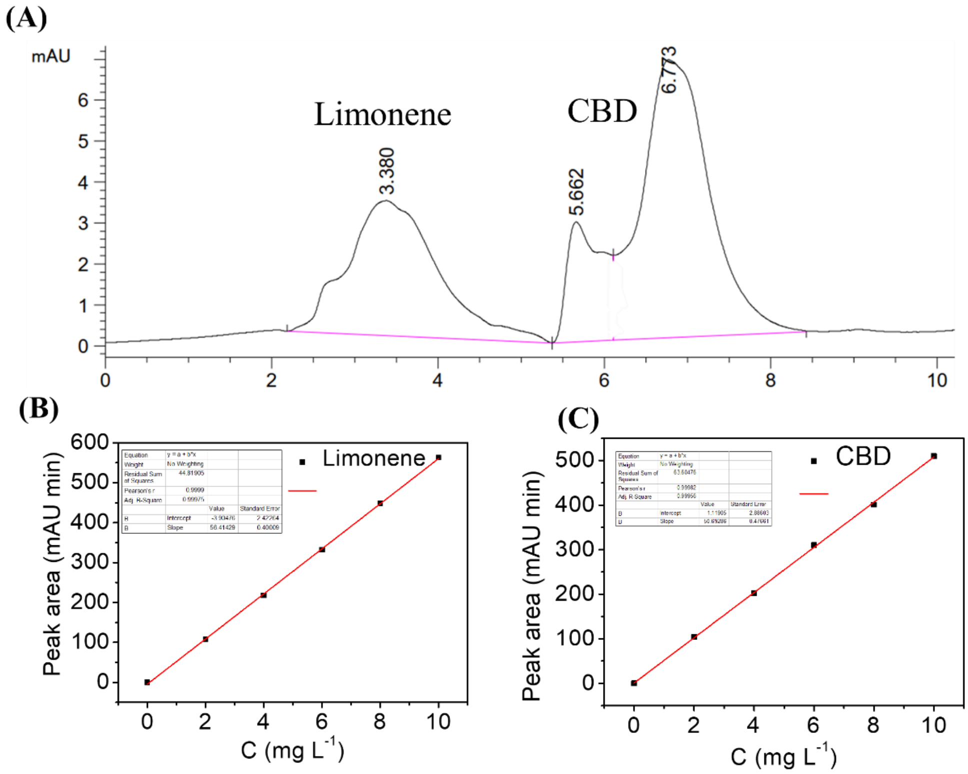

Fig. S32. (A) High-performance liquid chromatography (HPLC) of limonene and CBD. Standard curves of (B) limonene and (C) CBD by HPLC.

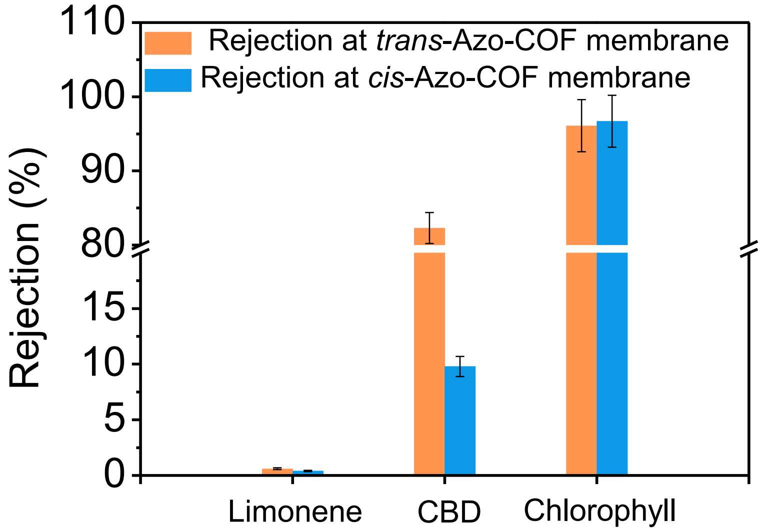

Fig. S33. Ternary system separation process of CBD and its competition molecules by the *trans/cis*-Azo-COF membranes.

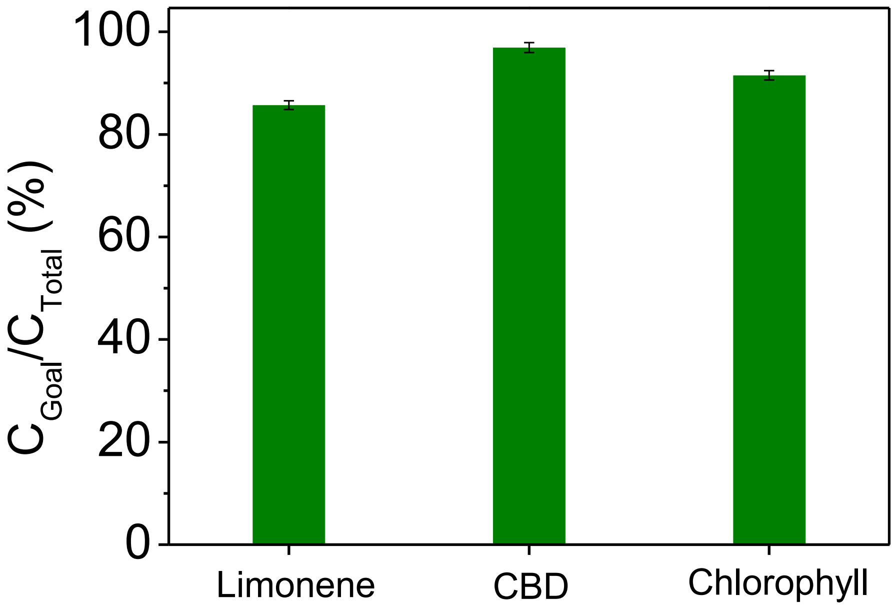

Fig. S34. Enrichment factor of CBD and its competition molecules.

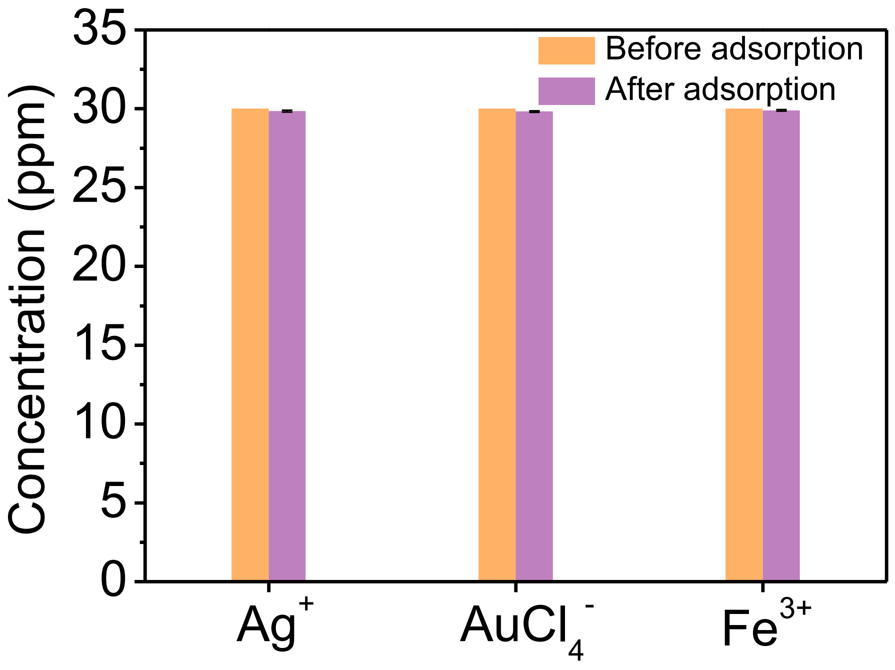

Fig. S35. The concentrations of different ions before and after the membrane was immersed in the feed solutions.

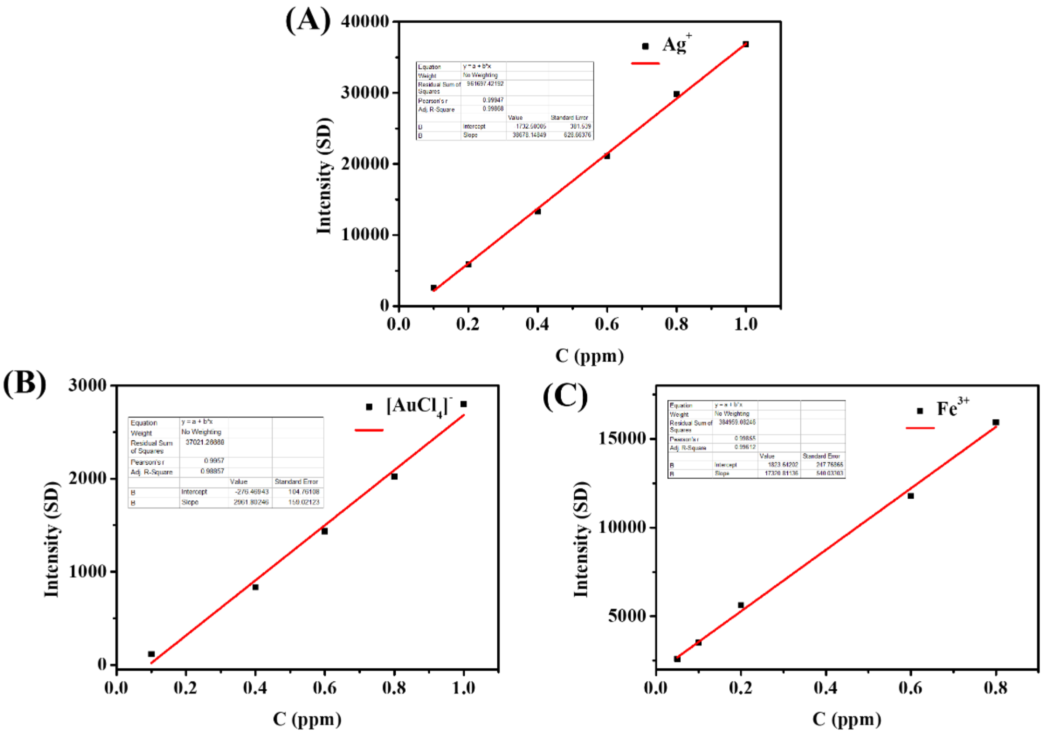

Fig. S36. Standard curves of metal ions in aqueous solution from ICP-OES measurements.

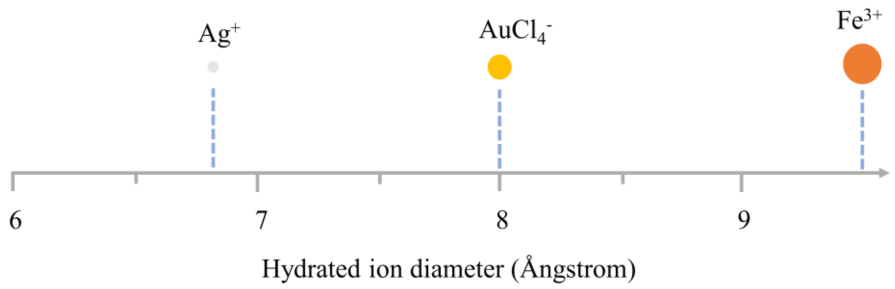

Fig. S37. Diameters of the hydrated ions.

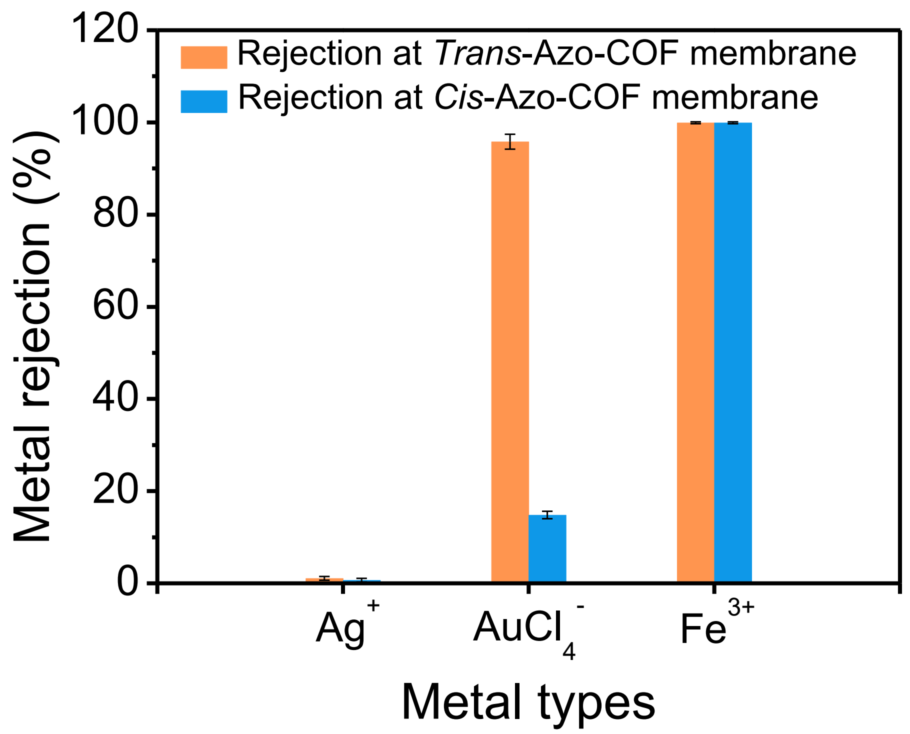

Fig. S38. The rejection rate of different ions in ternary system by the *trans/cis*-Azo-COF membranes.

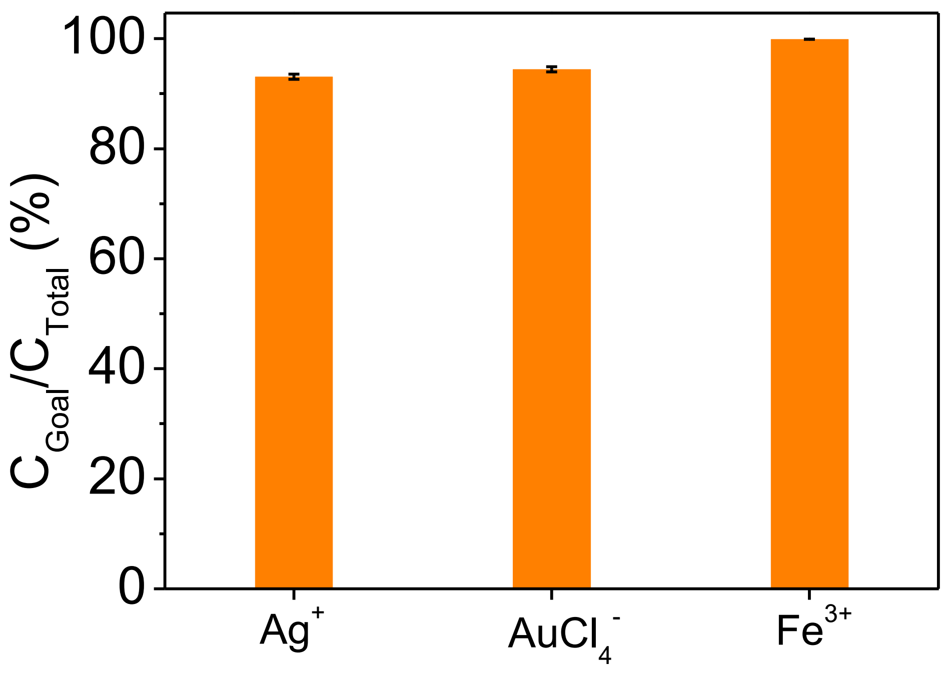

Fig. S39. Enrichment factor of different ions.

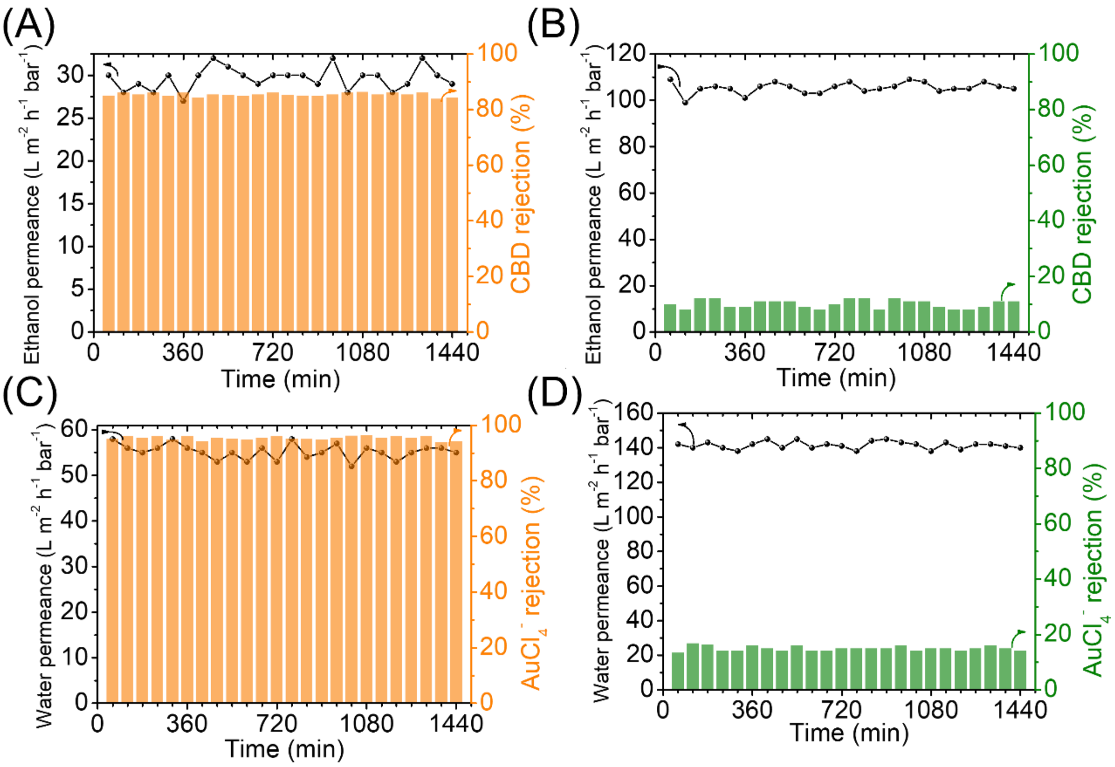

Fig. S40. CBD rejection and ethanol permeance of *trans*-Azo-COF membrane (A) and *cis*-Azo-COF membrane (B) under varied operation time. $AuCl_4^-$ rejection and water permeance of *trans*-Azo-COF membrane (C) and *cis*-Azo-COF membrane (D) under varied operation time.

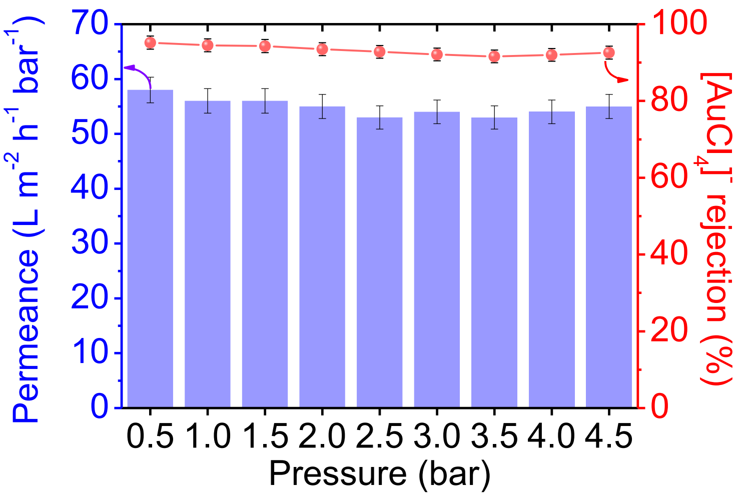

Fig. S41. The $AuCl_4^-$ rejection and water permeance of Azo-COF membrane under different pressure.

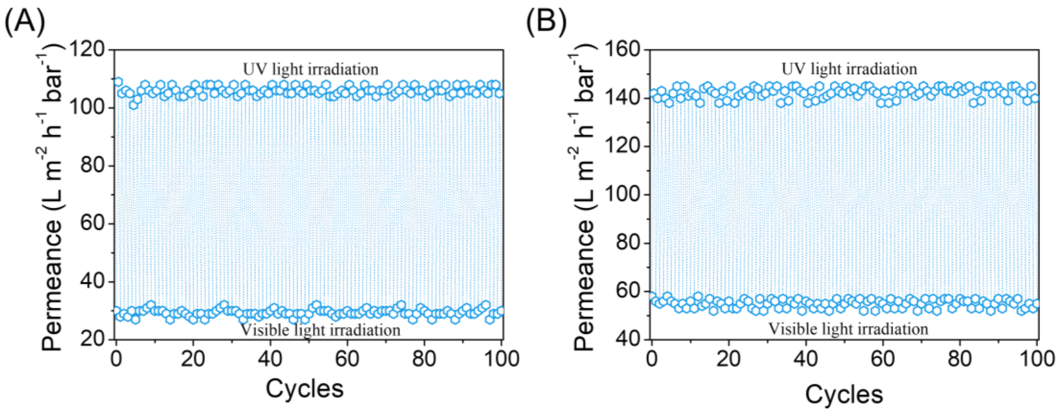

Fig. S42. (A) Switchable ethanol permeance upon the irradiation of alternating UV and visible light over 100 cycles. (B) Switchable water permeance upon the irradiation of alternating UV and visible light over 100 cycles.

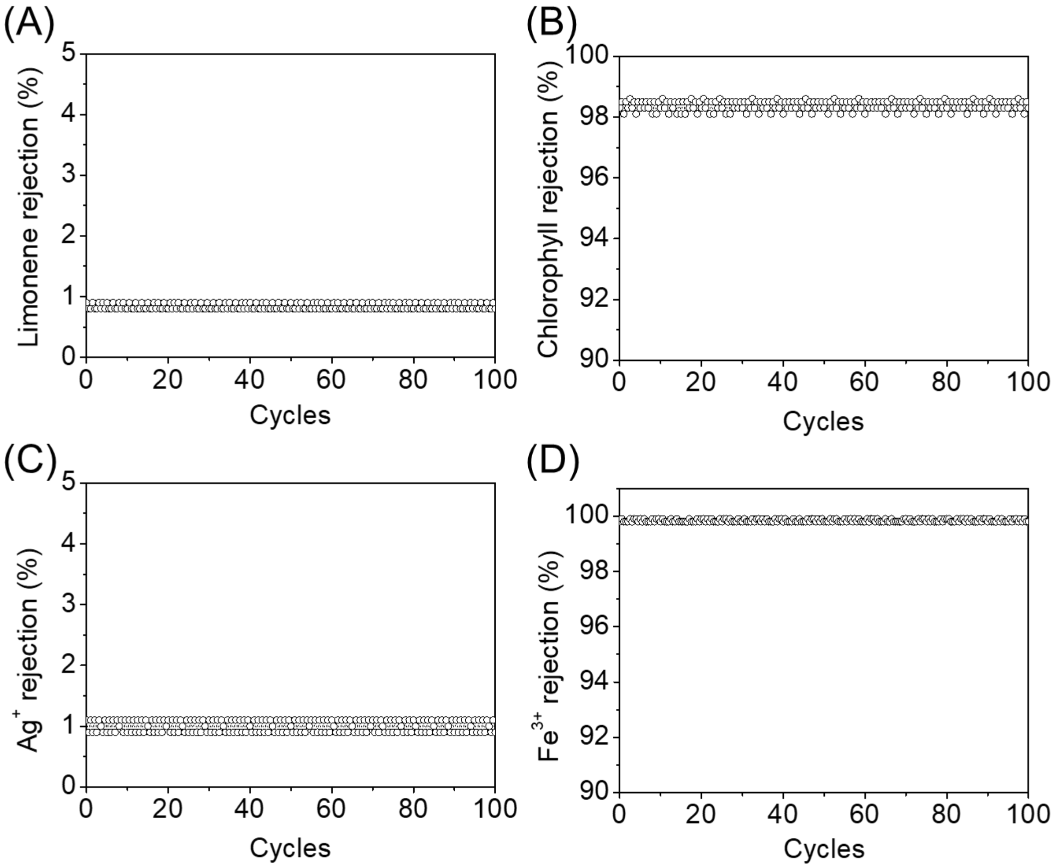

Fig. S43. Switchable of (A) limonene and (B) chlorophyll rejection of the COF membranes upon the irradiation of alternative UV and visible light for 100 cycles. Switchable of (C) Ag^{+} and (D) Fe^{3+} rejection of the COF membranes upon the irradiation of alternative UV and visible light for 100 cycles.

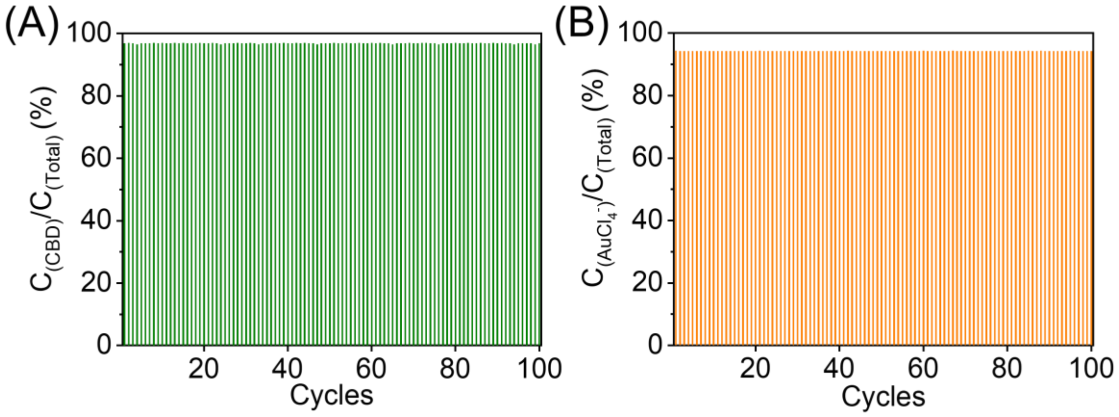

Fig. S44. The enrichment factors of CBD and $AuCl_4^-$ over 100 cycles.

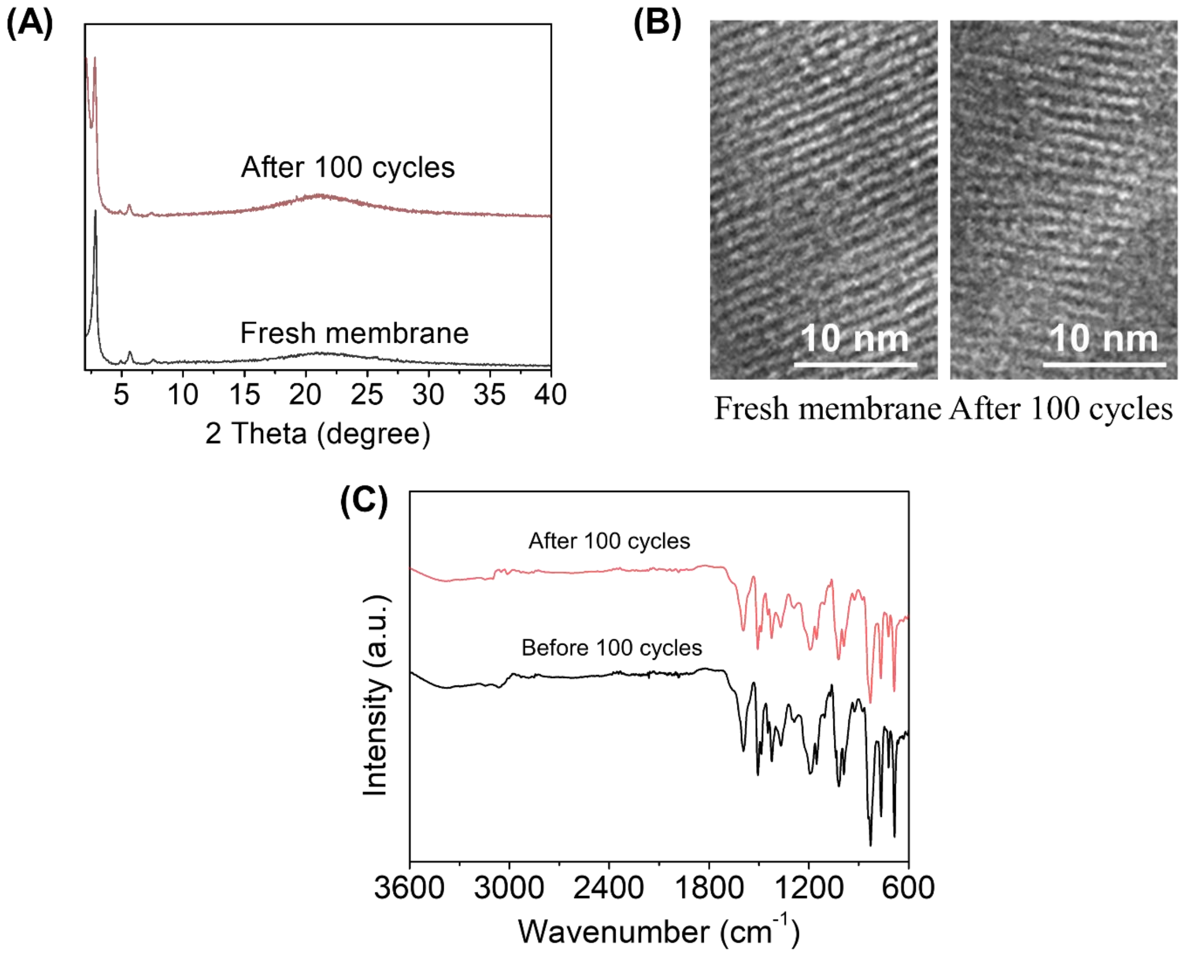

Fig. S45. (A)PXRD patterns of the Azo-COF membranes before and after 100 cycles. (B) HRTEM imagines of the Azo-COF membranes before and after 100 cycles. (C) The FT-IR spectra of Azo-COF membrane after 100 cycles.

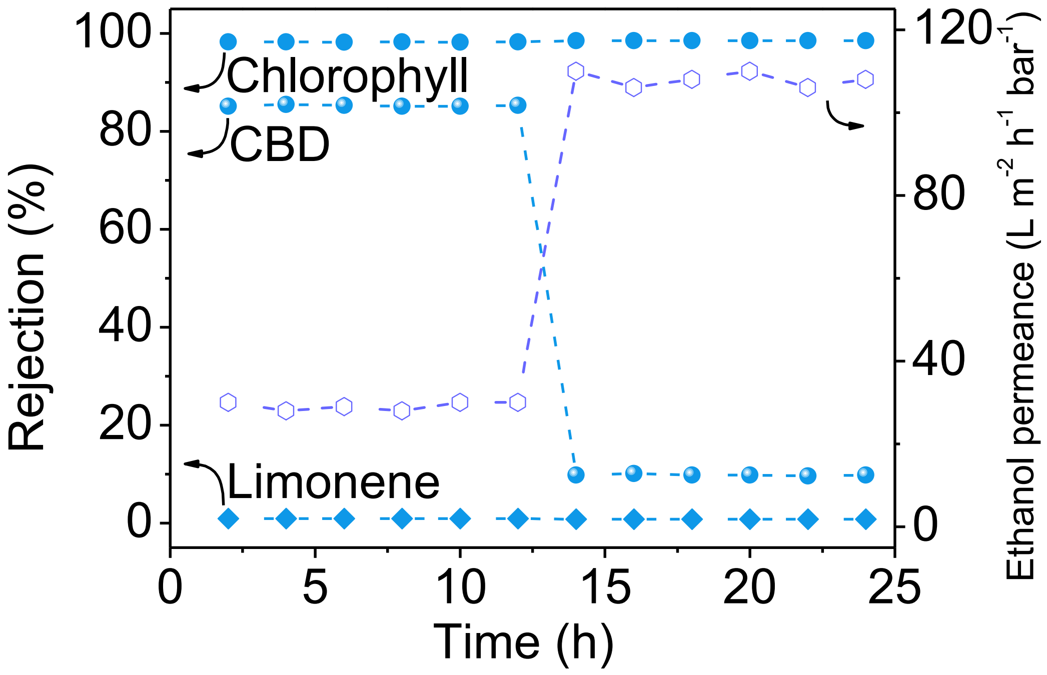

Fig. S46. Ethanol permeance and the rejection of limonene, CBD and chlorophyll by the Azo-COF membrane within 24 h operation.

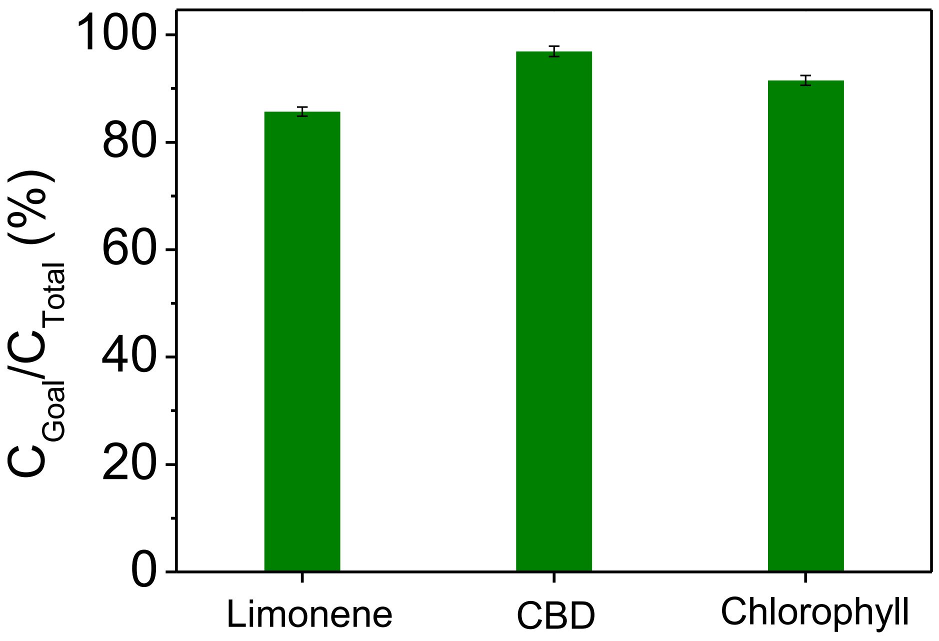

Fig. S47. Enrichment factor of limonene, CBD and chlorophyll after *trans-cis* two-step process.

3.Tables S1-S4

Table S1. Elemental analysis data of TAPB-BPTA COF and Azo-COF membranes

	C(%)	H(%)	N(%)	y(wt%)
COF membrane	71.51	4.36	5.79	-
Azo-COF membrane	60.48 [a]	3.82 [a]	16.44 [a]	53.7
	58.56 [b]	3.65 [b]	14.04 [b]	53.5

[a] Theoretical value: corresponding to a reaction of ~100% alkyne groups in the COF membrane with azobenzene groups; [b] Experiment value.

The molecular grafting rate (y) values were calculated based on the elemental analysis data according to the procedure reported in literature (*16*).

Table S2. The atomic percentage of C, N and O in the Azo-COF membrane from different testing technologies

	C (atom%)	N (atom%)	O (atom%)
Elemental analysis	82.1%	15.3%	2.6%
XPS	81.6%	14.1%	4.3%
EDS	82.7%	14.6%	2.7%

Table S3. The comparison of CBD / limonene quantification results by UV-vis spectroscopy and HPLC

	Trans state		*Cis* state	
	UV-Vis	HPLC	UV-Vis	HPLC
Limonene	0.9%	0.6%	0.8%	0.4%
CBD	85.3%	82.2%	8.9%	9.8%

Table S4. The metal ions concentrations at the permeate side through *trans/cis*-Azo COF membrane

	Ag^+ (ppm)	$[AuCl_4]^-$ (ppm)	Fe^{3+} (ppm)
Trans-Azo-COF membranes [a]	29.68	1.26	0.98
cis-Azo-COF membranes [b]	-	25.56	1.1

[a] For the permeation of *trans*-Azo-COF membrane, only Ag^+ could permeate the membrane while $[AuCl_4]^-$ and Fe^{3+} were rejected at the feed side. [b] For the permeation of *cis*-Azo-COF membrane, $[AuCl_4]^-$ could permeate the membrane while and Fe^{3+} was still rejected.

Legend of Supplementary Data S1.

Supplementary Data S1. Raw powder X-ray diffraction (PXRD) data presenting azobenzene-grafted COF membrane compared to the pristine membrane shown in Figure 2E.

REFERENCES

1. F. Fuso Nerini, J. Tomei, L. S. To, I. Bisaga, P. Parikh, M. Black, A. Borrion, C. Spataru, V. Castán Broto, G. Anandarajah, B. Milligan, Y. Mulugetta, Mapping synergies and trade-offs between energy and the sustainable development goals. *Nat. Energy* **3**, 10–15 (2017).

2. C. Chung, J. Kim, B. K. Sovacool, S. Griffiths, M. Bazilian, M. Yang, Decarbonizing the chemical industry: A systematic review of sociotechnical systems, technological innovations, and policy options. *Energy Res. Soc. Sci.* **96**, 102955 (2023).

3. Y. Wan, M. Zheng, W. Yan, J. Zhang, R. Lv, Fundamentals and rational design of heterogeneous C-N coupling electrocatalysts for urea synthesis at ambient conditions. *Adv. Energy Mater.* **14**, 2303588 (2024).

4. S. Zhang, Y. He, Z. Zhang, C. Zhong, Machine learning aided investigation on the structure-performance correlation of MOF for membrane-based He/H_2 separation. *Green Chem. Eng.* **5**, 526–532 (2024).

5. X.-F. Hu, T. Jiang, H. Fan, Y.-F. Guan, J.-J. Chen, H.-Q. Yu, M. Elimelech, Dual-regulated covalent organic framework membranes with near-theoretical pore sizes for angstrom-scale ion separations. *Sci. Adv.* **11**, eady3587 (2025).

6. J. Zhang, B. Liu, C. Chen, S. Jiang, Y. Zhang, B. Xu, A. Li, J. Xu, D. Wang, L. Zhang, Y. Hu, J. Li, D. Wu, J. Chu, Z. Shen, Ultrafast laser-ablated bioinspired hydrogel-based porous gating system for sustained drug release. *ACS Appl. Mater. Interfaces* **14**, 35366–35375 (2022).

7. A. M. Hetherington, F. I. Woodward, The role of stomata in sensing and driving environmental change. *Nature* **424**, 901–908 (2003).

8. Z. Mahimwalla, K. G. Yager, J.-i. Mamiya, A. Shishido, A. Priimagi, C. J. Barrett, Azobenzene photomechanics: Prospects and potential applications. *Polym. Bull.* **69**, 967–1006 (2012).

9. Z. Wang, A. Knebel, S. Grosjean, D. Wagner, S. Brase, C. Woll, J. Caro, L. Heinke, Tunable molecular separation by nanoporous membranes. *Nat. Commun.* **7**, 13872 (2016).

10. J. Liu, S. Wang, T. Huang, P. Manchanda, E. Abou-Hamad, S. P. Nunes, Smart covalent organic networks (CONs) with "on-off-on" light-switchable pores for molecular separation. *Sci. Adv.* **6**, eabb3188 (2020).

11. Y. Fu, Y. Gao, H. Jia, Y. Zhao, Y. Feng, W. Zhu, F. Zhang, M. D. Argyle, M. Fan, Rational engineering of triazine-benzene linked covalent-organic frameworks for efficient CO_2 photoreduction. *Green Energy Environ.* **10**, 804–812 (2025).

12. Y. Shao, R. Shen, S. Wang, S. Li, P. Zhang, X. Li, Composition engineering in covalent organic frameworks for tailored photocatalysis. *Acta Phys. Chim. Sin.* **41**, 100176 (2025).

13. C. Yin, Z. Zhang, Z. Si, X. Shi, Y. Wang, Smart covalent organic frameworks with intrapore azobenzene groups for light-gated ion transport. *Chem. Mater.* **34**, 9212–9220 (2022).

14. L. Ren, J. Chen, J. Han, J. Liang, H. Wu, Biomimetic construction of smart nanochannels in covalent organic framework membranes for efficient ion separation. *Chem. Eng. J.* **482**, 148907 (2024).

15. X. Jing, M. Zhang, Z. Mu, P. Shao, Y. Zhu, J. Li, B. Wang, X. Feng, Gradient channel segmentation in covalent organic framework membranes with highly oriented nanochannels. *J. Am. Chem. Soc.* **145**, 21077–21085 (2023).

16. S. Gao, Z. Li, Y. Yang, Z. Wang, Y. Wang, S. Luo, K. Yao, J. Qiu, H. Wang, L. Cao, Z. Lai, J. Wang, The ionic liquid-H_2O interface: A new platform for the synthesis of highly crystalline and molecular sieving covalent organic framework membranes. *ACS Appl. Mater. Interfaces* **13**, 36507–36516 (2021).

17. S. Gao, Q. Zhang, X. Su, X. Wu, X.-G. Zhang, Y. Guo, Z. Li, J. Wei, H. Wang, S. Zhang, J. Wang, Ingenious artificial leaf based on covalent organic framework membranes for boosting CO_2 photoreduction. *J. Am. Chem. Soc.* **145**, 9520–9529 (2023).

18. J. Guo, Y. Xu, S. Jin, L. Chen, T. Kaji, Y. Honsho, M. A. Addicoat, J. Kim, A. Saeki, H. Ihee, S. Seki, S. Irle, M. Hiramoto, J. Gao, D. Jiang, Conjugated organic framework with three-

dimensionally ordered stable structure and delocalized π clouds. *Nat. Commun.* **4**, 2736 (2013).

19. S. Gao, L. Guo, W. Wan, Z. Li, H. Wang, S. Luo, J. Wang, Covalent organic framework membrane with angstrom discrimination in pore size for highly permselective ionic liquid nanofiltration. *ACS Sustain. Chem. Eng.* **11**, 15910–15918 (2023).

20. F. Haase, K. Gottschling, L. Stegbauer, L. S. Germann, R. Gutzler, V. Duppel, V. S. Vyas, K. Kern, R. E. Dinnebier, B. V. Lotsch, Tuning the stacking behaviour of a 2D covalent organic framework through non-covalent interactions. *Mater. Chem. Front.* **1**, 1354–1361 (2017).

21. M. Feng, Z. Niu, C. Xing, Y. Jin, X. Feng, Y. Zhang, B. Wang, Covalent organic framework based crosslinked porous microcapsules for enzymatic catalysis. *Angew. Chem. Int. Ed. Engl.* **62**, e202306621 (2023).

22. Y. Chen, J. Qiu, X.-G. Zhang, H. Wang, W. Yao, Z. Li, Q. Xia, G. Zhu, J. Wang, A visible light/heat responsive covalent organic framework for highly efficient and switchable proton conductivity. *Chem. Sci.* **13**, 5964–5972 (2022).

23. K. Li, D. Mei, Y.-s. Liu, B. Yan, Three-dimensional covalent organic frameworks containing diverse nitrogen sites for gold adsorption. *Chem. Mater.* **37**, 2535–2545 (2025).

24. Y. Zhao, X. Tao, B. Xu, W. Liu, S. Lin, Robust thiazole-linked covalent organic frameworks with post-modified azobenzene groups: Photo-regulated dye adsorption and separation. *Adv. Funct. Mater.* **34**, 2401895 (2024).

25. Q. Xu, S. Tao, Q. Jiang, D. Jiang, Designing covalent organic frameworks with a tailored ionic interface for ion transport across one-dimensional channels. *Angew. Chem. Int. Ed. Engl.* **59**, 4557–4563 (2020).

26. G. Zhang, Y.-l. Hong, Y. Nishiyama, S. Bai, S. Kitagawa, S. Horike, Accumulation of glassy poly(ethylene oxide) anchored in a covalent organic framework as a solid-state Li^+ electrolyte. *J. Am. Chem. Soc.* **141**, 1227–1234 (2018).

27. Y. Zhu, W. Zhang, Reversible tuning of pore size and CO_2 adsorption in azobenzene functionalized porous organic polymers. *Chem. Sci.* **5**, 4957–4961 (2014).

28. B. Sutariya, S. Karan, A realistic approach for determining the pore size distribution of nanofiltration membranes. *Sep. Purif. Technol.* **293**, 121096 (2022).

29. K. Xu, Y. Zheng, J. Zhou, Y. Zhao, X. Pang, L. Cheng, H. Wang, X. Zhang, R. Zhang, Z. Jiang, Microwave-assisted fabrication of highly crystalline, robust COF membrane for organic solvent nanofiltration. *Adv. Funct. Mater.* **35**, 2417383 (2025).

30. S. Gao, Y. Yang, L. Zhai, Y. Zhao, Z. Li, H. Wang, J. Wang, Alkoxy-functionalized covalent organic framework membranes for efficient molecular sieving with high water permeance. *Sep. Purif. Technol.* **356**, 129903 (2025).

31. X. Tian, X. Zhao, Z. Wang, Y. Shi, Z. Li, J. Qiu, H. Wang, S. Zhang, J. Wang, Efficient capture and low energy release of NH_3 by azophenol decorated photoresponsive covalent organic frameworks. *Angew. Chem. Int. Ed. Engl.* **63**, e202406855 (2024).

32. G. Das, T. Prakasam, M. A. Addicoat, S. K. Sharma, F. Ravaux, R. Mathew, M. Baias, R. Jagannathan, M. A. Olson, A. Trabolsi, Azobenzene-equipped covalent organic framework: Light-operated reservoir. *J. Am. Chem. Soc.* **141**, 19078–19087 (2019).

33. S. Pisanti, A. M. Malfitano, E. Ciaglia, A. Lamberti, R. Ranieri, G. Cuomo, M. Abate, G. Faggiana, M. C. Proto, D. Fiore, C. Laezza, M. Bifulco, Cannabidiol: State of the art and new challenges for therapeutic applications. *Pharmacol. Ther.* **175**, 133–150 (2017).

34. C. Y. Schluttenhofer, L. Yuan, Hemp hemp hooray for cannabis research. *Science* **363**, 701–702 (2019).

35. A. Dobrinčić, M. Repajić, I. E. Garofulić, L. Tuđen, V. Dragović-Uzelac, B. Levaj, Comparison of different extraction methods for the recovery of olive leaves polyphenols. *Processes* **8**, 1008 (2020).

36. T. M. Attard, C. Bainier, M. Reinaud, A. Lanot, S. J. McQueen-Mason, A. J. Hunt, Utilisation of supercritical fluids for the effective extraction of waxes and cannabidiol (CBD) from hemp wastes. *Ind. Crops Prod.* **112**, 38–46 (2018).

37. J. A. A. Grimm, H. Zhou, R. Properzi, M. Leutzsch, G. Bistoni, J. Nienhaus, B. List, Catalytic asymmetric synthesis of cannabinoids and menthol from neral. *Nature* **615**, 634–641 (2023).

38. J. Qiu, C. Xu, X. Xu, Y. Zhao, Y. Zhao, Y. Zhao, J. Wang, Porous covalent organic framework based hydrogen-bond nanotrap for the precise recognition and separation of gold. *Angew. Chem. Int. Ed. Engl.* **62**, e202300459 (2023).

39. W. Wang, M. Wu, W. Xue, X. Zhao, Z. Fang, L. Nie, Y. Heng, H. Huang, C. Zhong, A robust cationic covalent triazine framework-based nanotrap for fast and efficient recovery of gold from electronic waste. *Chem. Eng. J.* **483**, 149208 (2024).

40. E. Mohammadi, M. Pourabdoli, M. Ghobeiti-Hasab, A. Heidarpour, Ammoniacal thiosulfate leaching of refractory oxide gold ore. *Int. J. Miner. Process.* **164**, 6–10 (2017).

41. I. N. Rizki, Y. Tanaka, N. Okibe, Thiourea bioleaching for gold recycling from e-waste. *Waste Manag.* **84**, 158–165 (2019).

42. Y. Guo, X. Guo, H. Wu, S. Li, G. Wang, X. Liu, G. Qiu, D. Wang, A novel bio-oxidation and two-step thiourea leaching method applied to a refractory gold concentrate. *Hydrometallurgy* **171**, 213–221 (2017).

43. B. Lin, W. Chen, Y. Lei, X. Ma, J. Wang, L. Li, Solvothermal preparation of microporous polyureas for Au(III) adsorption. *Langmuir* **40**, 9001–9011 (2024).

44. M. Liu, D. Jiang, Y. Fu, G. Zheng Chen, S. Bi, X. Ding, J. He, B. H. Han, Q. Xu, G. Zeng, Modulating skeletons of covalent organic framework for high-efficiency gold recovery. *Angew. Chem. Int. Ed. Engl.* **63**, e202317015 (2023).

45. Y. Wang, L. Xiao, H. Liu, P. Qian, S. Ye, Y. Chen, Acid leaching pretreatment on two-stage roasting pyrite cinder for gold extraction and co-precipitation of arsenic with iron. *Hydrometallurgy* **179**, 192–197 (2018).

46. Y. Qiang, S. Gao, Y. Zhang, S. Wang, L. Chen, L. Mu, H. Fang, J. Jiang, X. Lei, Thermally reduced graphene oxide membranes revealed selective adsorption of gold ions from mixed ionic solutions. *Int. J. Mol. Sci.* **24**, 12239 (2023).

47. Y. Marcus, Thermodynamics of solvation of ions. Part 5.—Gibbs free energy of hydration at 298.15 K. *J. Chem. Soc. Faraday Trans.* **87**, 2995–2999 (1991).

48. K. Wang, X. Qiao, H. Ren, Y. Chen, Z. Zhang, Industrialization of covalent organic frameworks. *J. Am. Chem. Soc.* **147**, 8063–8082 (2025).

49. J. E. Chen, Z. J. Yang, H. U. Koh, J. Shen, Y. Cai, Y. Yamauchi, L. H. Yeh, V. Tung, K. C. W. Wu, Current progress and scalable approach toward the synthesis of 2D metal–organic frameworks. *Adv. Mater. Interfaces* **9**, 2102560 (2022).

50. M. Parkinson, H. Vardhan, R. Verduzco, J. Fortner, M. Elimelech, Toward continuous, oriented covalent organic framework membranes for precise molecular separations. *ACS Nano* **19**, 29934–29960 (2025).

51. Y. Wang, H. Wang, Y. Liu, M. Peng, H. Fan, H. Meng, A comprehensive review on the scalable and sustainable synthesis of covalent organic frameworks. *Chin. Chem. Lett.* **36**, 110189 (2025).

52. K. V. Kutonova, M. E. Trusova, P. S. Postnikov, V. D. Filimonov, J. Parello, A simple and effective synthesis of aryl azides via arenediazonium tosylates. *Synthesis* **45**, 2706–2710 (2013).

53. Z. Wojnarowska, E. Thoms, B. Blanchard, S. N. Tripathy, P. Goodrich, J. Jacquemin, J. Knapik-Kowalczuk, M. Paluch, How is charge transport different in ionic liquids? The effect of high pressure. *Phys. Chem. Chem. Phys.* **19**, 14141–14147 (2017).

54. R. Wang, X. Shi, Z. Zhang, A. Xiao, S. Sun, Z. Cui, Y. Wang, Unidirectional diffusion synthesis of covalent organic frameworks (COFs) on polymeric substrates for dye separation. *J. Membr. Sci.* **586**, 274–280 (2019).

55. L. Wang, J. Lin, J. Ye, Y. Lim, C. Chen, C. Dong, T. Liu, Enrichment of persistent organic pollutants in microplastics from coastal waters. *Environ. Sci. Technol.* **58**, 22391–22404 (2024).

56. Z. Jiang, R. Dong, A. M. Evans, N. Biere, M. A. Ebrahim, S. Li, D. Anselmetti, W. R. Dichtel, A. G. Livingston, Aligned macrocycle pores in ultrathin films for accurate molecular sieving. *Nature* **609**, 58–64 (2022).

57. C. Yin, M. Liu, Z. Zhang, M. Wei, X. Shi, Y. Zhang, J. Wang, Y. Wang, Perpendicular alignment of covalent organic framework (COF) pore channels by solvent vapor annealing. *J. Am. Chem. Soc.* **145**, 11431–11439 (2023).

58. B. Mishra, B. P. Tripathi, Flexible covalent organic framework membranes with linear aliphatic amines for enhanced organic solvent nanofiltration. *J Mater Chem A* **11**, 16321–16333 (2023).
