# Supplementary figures and images for: Highly efficient light-gated COF membrane for precise multistage molecular separation
Source: Sci Adv. 2026 Mar 13;12(11):eadz1929. doi: 10.1126/sciadv.adz1929 (PMC12985721; doi:10.1126/sciadv.adz1929)

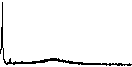

Supplement: Supplementary file 3 — Data S1 [file sciadv.adz1929_data_s1.zip › Azo-COF membrane.jip]

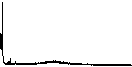

Supplement: Supplementary file 3 — Data S1 [file sciadv.adz1929_data_s1.zip › COF membrane.jip]
